# Physical plasma-treated saline promotes an immunogenic phenotype in CT26 colon cancer cells in vitro and in vivo

**DOI:** 10.1038/s41598-018-37169-3

**Published:** 2019-01-24

**Authors:** Eric Freund, Kim Rouven Liedtke, Julia van der Linde, Hans-Robert Metelmann, Claus-Dieter Heidecke, Lars-Ivo Partecke, Sander Bekeschus

**Affiliations:** 1 ZIK plasmatis, Leibniz Institute for Plasma Science and Technology (INP Greifswald), Felix-Hausdorff-Str. 2, 17489 Greifswald, Germany; 2 Department of General, Visceral, Thoracic and Vascular Surgery, Greifswald University Medical Center, Ferdinand-Sauerbruch-Str., 17475 Greifswald, Germany; 3 Oral and Maxillofacial Surgery/Plastic Surgery, Greifswald University Medical Center, Ferdinand-Sauerbruch-Str., 17475 Greifswald, Germany

## Abstract

Metastatic colorectal cancer is the fourth most common cause of cancer death. Current options in palliation such as hyperthermic intraperitoneal chemotherapy (HIPEC) present severe side effects. Recent research efforts suggested the therapeutic use of oxidant-enriched liquid using cold physical plasma. To investigate a clinically accepted treatment regimen, we assessed the antitumor capacity of plasma-treated saline solution. In response to such liquid, CT26 murine colon cancer cells were readily oxidized and showed cell growth with subsequent apoptosis, cell cycle arrest, and upregulation of immunogenic cell death (ICD) markers *in vitro*. This was accompanied by marked morphological changes with re-arrangement of actin fibers and reduced motility. Induction of an epithelial-to-mesenchymal transition phenotype was not observed. Key results were confirmed in MC38 colon and PDA6606 pancreatic cancer cells. Compared to plasma-treated saline, hydrogen peroxide was inferiorly toxic in 3D tumor spheroids but of similar efficacy in 2D models. *In vivo*, plasma-treated saline decreased tumor burden in Balb/C mice. This was concomitant with elevated numbers of intratumoral macrophages and increased T cell activation following incubation with CT26 cells *ex vivo*. Being a potential adjuvant for HIPEC therapy, our results suggest oxidizing saline solutions to inactivate colon cancer cells while potentially stimulating antitumor immune responses.

## Introduction

Despite continuous advances in medicine, therapy and palliation of peritoneal colon cancer metastasis remains challenging^1^. This owes to the high number of sometimes difficult to visualize tumor nodes in the peritoneum that hinder surgical resection^2^. Early therapeutic options are limited because peritoneal metastasis often occurs before primary diagnosis of colon cancer^3^. As consequence, rapid tumor spread and infiltration into multiple organs are main factors constituting to the bad prognosis in many patients^4^. Current therapeutic choices for peritoneal metastasis from colorectal cancer involve classical chemotherapy, irradiation, cytoreductive surgery, and hyperthermic intraperitoneal chemotherapy (HIPEC)^5^. Yet, many cancers acquire chemoresistance and numerous patients ultimately do not benefit from therapy^6^. If successful, therapeutic profit often comes with substantial side effects^7^. This clinical situation calls for new roads for (adjuvant and palliative) therapeutic targeting of metastatic colorectal cancer. Among others, one route is sensitizing colorectal cancer via modulation of the antioxidant defense mechanisms important for handling reactive oxygen and nitrogen species (ROS/RNS)^8–10^.

A recent innovation in prospective cancer therapy is cold physical plasma, a partially ionized gas that produces a plethora of ROS/RNS^11–13^. Previous studies have provided evidence for tumor-toxic effects of cold plasma in colorectal cancers *in vitro*^14–16^. The proof-of-concept was further validated in a non-orthotopic murine model of human colon cancer *in vivo*, and effects were delineated to generation of reactive species^17^. The utilization of liquid previously exposed to plasmas surprisingly paralleled many of the antitumor effects seen with direct plasma treatment^18–20^. As carrier liquid, cell culture medium is often used to target tumor cells^21–23^. This approach has also successfully decreased tumor-burden in mice with widespread peritoneal pancreatic or gastric cancer metastasis^24,25^. While these studies are promising, the multi-component cell culture medium is unlikely to receive accreditation for use in clinics due to its complex formulation and unknown immunogenicity. By contrast, saline solutions are clinically accepted and utilized worldwide. Therefore, the aim of this study was to investigate the tumor-toxic and pro-immunogenic properties of physical plasma-treated saline on murine colorectal cancer cells *in vitro*.

During microevolution in hosts, various cancer cell types with high turnover rates develop mechanisms to escape immunosurveillance^26–29^. One approach of cancer therapy is to utilize the immunogenic cancer cell death (ICD) triggering the immune system as defender against tumors. In this concept, antigen-presenting cells phagocytosing tumor material become activated through sensing of tumor cell surface marker or the release of soluble factors that act as damage-associated molecular patterns (DAMPs)^30–33^. This may generate an immune-response directed against these cancer cells. Hence, the emission of such DAMPs is accompanied with prognostic benefit in cancer patients^34^. We here evaluated the potential of plasma-treated saline that may serve as basis for a future solution used for peritoneal lavage to induce toxicity and immunogenicity in colon cancer cells. We found that such solution was storable over weeks, mediated not only cell death in tumor cells but also altered their phenotype in disadvantage of metastatic spread, and acted as a *bone fide* inducer of pro-immunogenic markers.

## Results

### Plasma-treated saline was toxic to 2D and 3D colon cancer cultures

**Figure 1:**
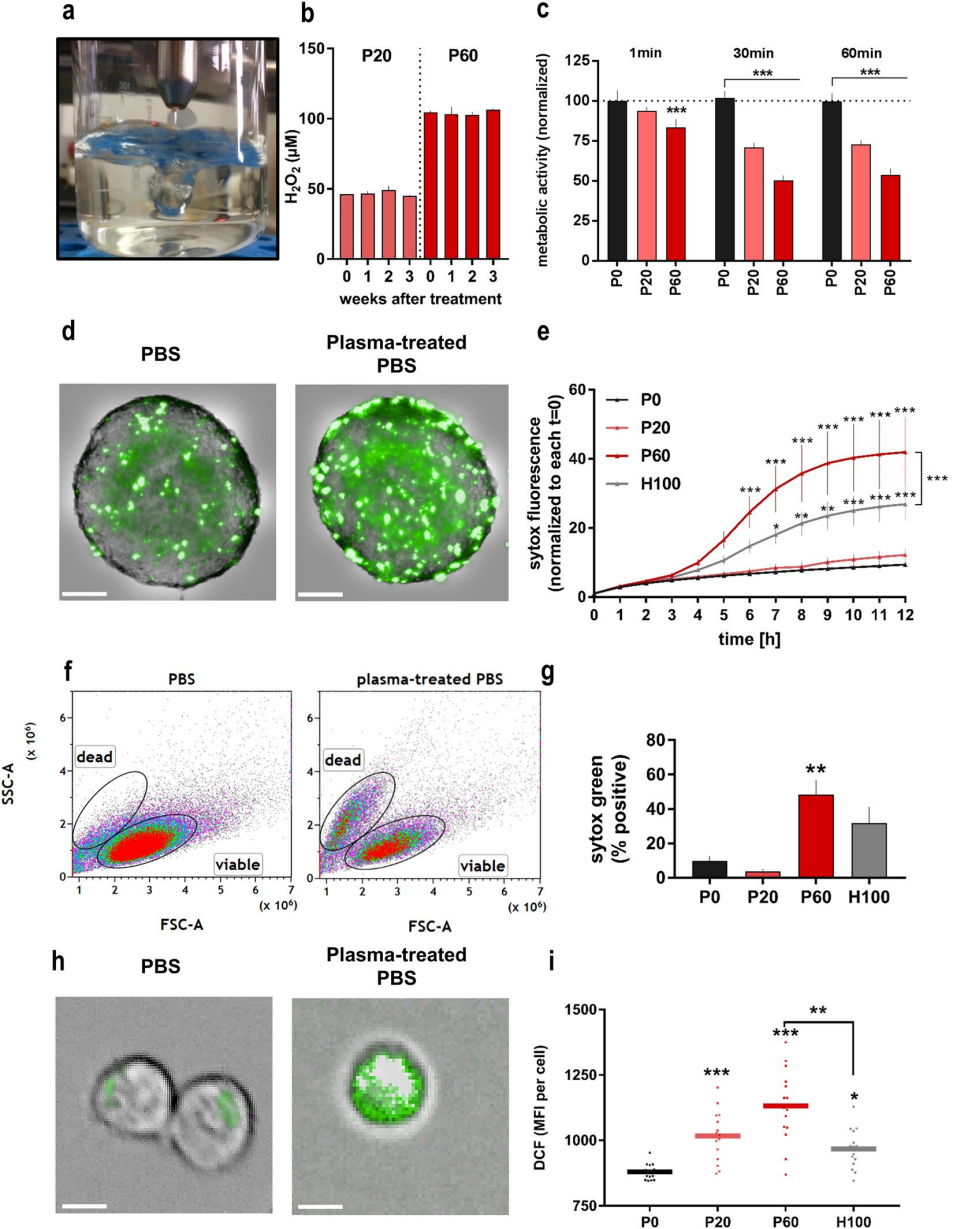
Plasma-treated saline contained hydrogen peroxide, and oxidized and inactivated cancer cells grown in 2D and 3D cultures. (**a**) Treatment of bulk phosphate-buffered saline (PBS) solution with the kINPen argon plasma jet; (**b**) measurement of hydrogen peroxide (H_2_O_2_) in PBS after repeated freeze-thaw cycles; (**c**) metabolic activity of CT26 cells after incubation with control and plasma-treated PBS for 1 min, 30 min, or 60 min (normalized on the control of 1 min exposure to control saline); (**d**) maximum intensity projection of representative brightfield and sytox green images of tumor spheroids (scale bar = 100 µm); (**e**) sytox green fluorescence during distinct time-points after exposure to saline solutions; flow cytometry measurement (**f**) and percentage of sytox green positive single cells (**g**), detached from spheroids; (**h**) representative overlay brightfield and fluorescence images of CM-H_2_DCFDA-labeled CT26 cells exposed to PBS or plasma-treated PBS (scale bar = 50 µm); (**i**) image quantification of nine fields of view in 4 replicates per condition of experiment depicted in (**h**). Data are presented as mean (**i**) and SD (**b**,**c**,**e**,**g**) of 2–3 independent experiments; statistical analysis was carried out with Wilcoxon rank test to compare P60 to H100 (**e**,**i**) or ANOVA (**c**,**e**,**g**,**i**); normalization was carried out to each P0 (**c**) or to each t = 0 (**e**); P0 = control PBS, P20 and P60 = plasma-treated PBS, H100 = concentration-matched hydrogen peroxide to P60.

In view of a possible application of a solution that can be used for *in vitro* experiments as well as for future peritoneal lavage, we treated 50 ml of phosphate-buffered saline (PBS) solution with an argon plasma jet to deposit plasma-derived oxidants (Fig. 1a). Exposure times were either 20 min (P20) or 60 min (P60) with subsequent compensation for evaporation with double distilled water. Deposition of hydrogen peroxide (H_2_O_2_) is common with argon plasma jets, and P60 correlated to a final concentration of 100 µM of H_2_O_2_. This concentration was measured constantly even after repeated freeze-thaw cycles of aliquots of this solution (Fig. 1b). In addition, the treatment regimen generated nitrate, nitrite, and superoxide (Supplementary Fig. S1) but not hypochlorous acid (data not shown) in saline solution. For cell experiments, four regimens were used for treatment of CT26 colorectal cancer cells: P0 (control PBS), P20, P60, and H100 (100 µM of experimentally added H_2_O_2_ into 50 ml of PBS that corresponds to the concentration of H_2_O_2_ generated with the P60 condition). Cell cultures need specialized media to meet their energy needs, which PBS does not. To test the optimal incubation time with our saline solutions, metabolic activity was assessed 24 h after treatment (Fig. 1c). Thirty minutes of incubation with P20 and P60 saline were more toxic compared to 1 min of incubation but similar efficient than 60 min. Therefore, the 30 min exposure time was chosen for subsequent experiments. Using the H_2_O_2_ scavenging enzyme catalase, we confirmed that H_2_O_2_ was mainly responsible for the cytotoxic effect of the P20 and P60 as well as the H100 treatment (Supplementary Fig. S2). The cytotoxic effect was confirmed and even more pronounced in MC38 colorectal cancer cells, and less pronounced in PDA6606 pancreatic cancer cells and HaCat keratinocytes (Supplementary Fig. S2). To test the tumor-toxic efficacy of plasma-treated saline in a physiologically more relevant model, cancer cell death was followed over 12 h post-exposure in a 3D tumor spheroids model (Fig. 1d). Quantitative image analysis from over 5,000 images revealed a significant increase in cell death with P60 and H100 exposure (Fig. 1e). Remarkably, plasma-treated saline (P60) was significantly more effective compared to H_2_O_2_ saline (H100). To confirm this finding, spheroids were collected 12 h after treatment, digested to single cell suspensions, and quantified for the percentage of dead cells using flow cytometry (Fig. 1f). Results confirmed plasma-treated but H_2_O_2_-supplemented saline had a significantly higher cytotoxic effect in 3D tumor spheroids compared to the control condition (Fig. 1g). To validate that this finding was related to oxidants deposited via plasma treatment and accumulating within cells, CT26 cells were labeled with chloromethyl 2′,7′-dichlorodihydrofluorescein diacetate (CM-H_2_DCF-DA), a redox-sensitive probe fluorescing upon intracellular oxidation^35^ with help of intracellular oxidases (Fig. 1h). High content imaging analysis of intracellular CM-H_2_DCF-DA mean fluorescence intensities (MFI) retrieved from several thousand cells per conditions revealed a significant increase in fluorescence for P20, P60, and H100 **(**Fig. 1i**)**. Corroborating findings with 3D tumor spheroids, plasma-treated saline (P60) gave a significantly stronger increase in fluorescence compared to the hydrogen peroxide-matched control condition H100. Despite the prime role of hydrogen peroxide in cytotoxicity as seen with catalase controls (Supplementary Fig. S2), this suggests plasma-derived oxidants other than H_2_O_2_ to play in role in oxidation and cytotoxicity in tumor cells.

### Oxidizing saline solutions reduced colon cancer cell growth and induced apoptosis

**Figure 2:**
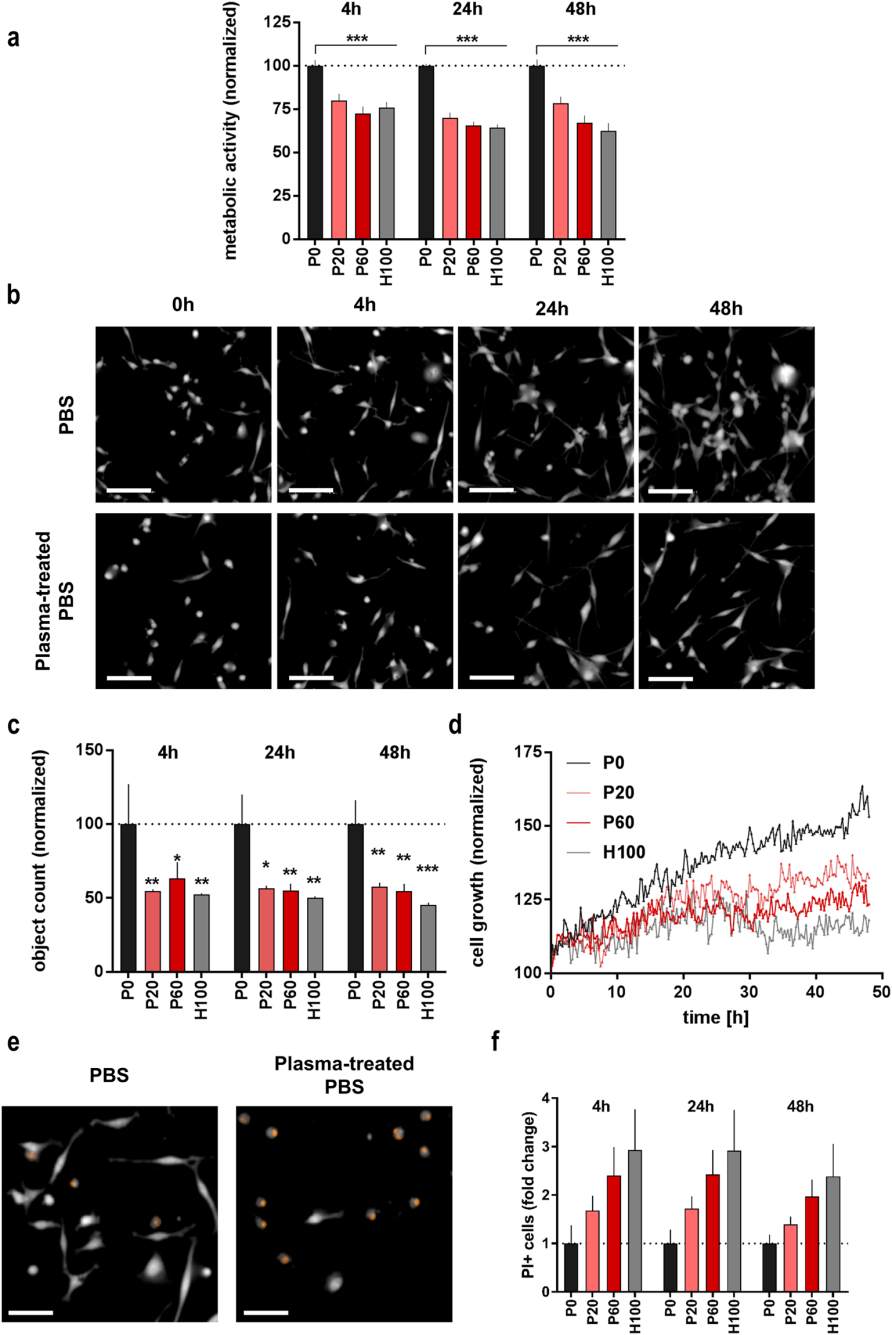
Plasma-treated saline decreased metabolic activity and cell growth of cancer cells. (**a**) Metabolic activity of cells incubated with saline solutions for 30 min after several time points; (**b**) representative images of the cytosolic fraction (imaging mode: digital phase contrast, DPC) of cells treated with PBS or plasma-PBS at several time points thereafter (scale bar = 150 µm); (**c**) image quantification of cell counts at several time points after exposure to saline solutions; (**d**) total cell growth area over 48 h in life cell imaging, each condition was normalized to its respective t = 0; imaging data are from of nine fields of view in 4 replicates per condition; (**e**) representative DPC and propidium iodide (PI) images (scale bar = 100 µm); (**f**) percent PI^+^ cells at 4 h, 24 h, and 48 h following exposure to saline solutions, imaging data are from of nine fields of view in 4 replicates per condition. Data are presented as mean and SEM of three independent experiments; statistical analysis was carried out with ANOVA; P0 = control PBS, P20 and P60 = plasma-treated PBS, H100 = concentration-matched hydrogen peroxide to P60. Data (**a**,**c,d,f**) were normalized to each P0 time point.

**Figure 3:**
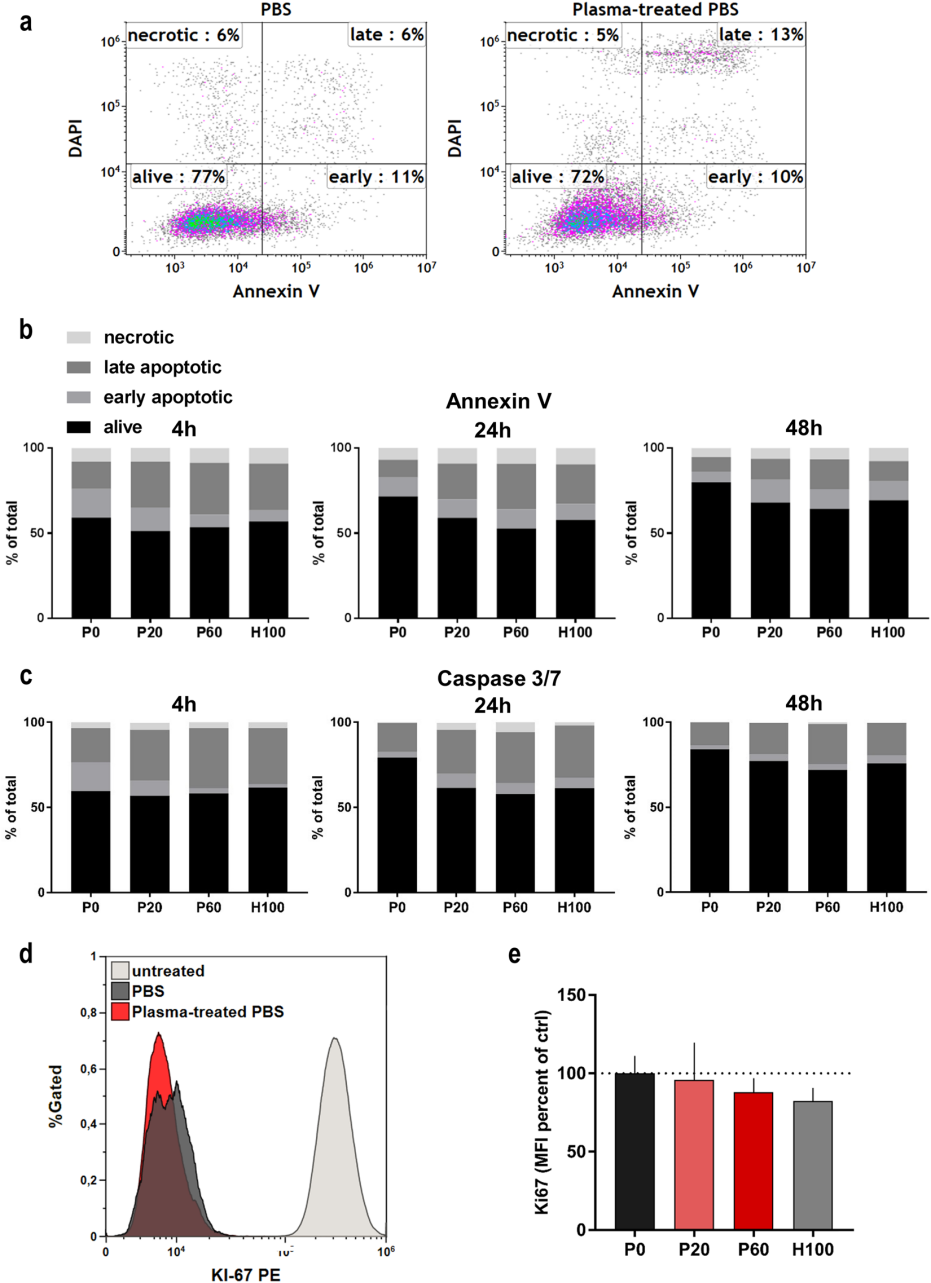
Plasma-treated saline modestly induced cell death in CT26 colon cancer cells. (**a**) Representative flow cytometry dot plots CT26 apoptosis measured via annexin V and DAPI 24 h after treatment with saline solutions; quantification of CT26 cell death via annexin V **(b**), Caspase 3/7 (**c**) and DAPI at different incubation times following exposure to saline solutions; (**d**) flow cytometry overlay histogram of fluorescence of antibody-labeled cell proliferation marker Ki67, untreated cells were not exposed to any saline; (**e**) quantitative analysis of Ki67 expression 4 h after treatment with saline solutions normalized to mean fluorescence intensity (MFI) of PBS control. Data are mean (SEM) of three independent experiments. Statistical analysis was carried out with ANOVA; P0 = control PBS, P20 and P60 = plasma-treated PBS, H100 = concentration-matched hydrogen peroxide to P60.

After 30 min of incubation with oxidizing saline solutions, a similar reduction in metabolic activity was observed after 4 h, 24 h, and 48 h when compared to individual P0 controls of each incubation time **(**Fig. 2a**)**. Despite the fact that our treatments were of moderate cytotoxicity (about 25%), this suggests that incubation with oxidizing saline solutions had a profound and long-lasting effect in CT26 colon cancer cells. To resolve *in vitro* growth kinetics in more depths, time-lapse video microscopy experiments were performed over 48 h. Digital phase contrast (DPC) images (Fig. 2b) were used to quantify the total number of object (Fig. 2c) and to calculate cytosolic area (Fig. 2d) in these kinetic experiments. With both parameter, a decrease was observed after exposure to plasma-treated as well as H_2_O_2_ saline. A decreased object count was also seen in MC38 cells to a higher extent, and in PDA6606 and HaCat cells to a lower extent (Supplementary Fig. 2) compared to CT26 cells (Fig. 2c). The next question was whether cell proliferation was reduced or whether cell death occurred. To investigate this, propidium iodide (PI) was used (Fig. 2e) to quantify terminal cell death (Fig. 2f). The relative increase of dead cells upon treatment to plasma-treated or H_2_O_2_ saline was consistent up to 48 h post exposure. To classify the mode of cell death, cells were stained with annexin V and 4′,6-diamidin-2-phenylindol (DAPI), and flow cytometry was utilized to distinguish between apoptotic and necrotic cells (Fig. 3a). Although small in amplitude, oxidizing saline solutions predominantly induced apoptosis with only few residual necrotic cells present (Fig. 3b). To confirm results with another apoptosis assays, samples were stained with a probe indicating caspase 3/7 activity in cells. Compared to annexin V experiments, similar rates of apoptosis were identified when staining for caspase activity (Fig. 3c). Cytotoxic conditions not only induce cell death but also may reduce proliferation. Hence, we stained for the cell proliferation marker Ki-67 (Fig. 3d). A modest, non-significant decrease in cell proliferation was observed (Fig. 3e).

### Oxidizing saline solutions promoted a morphologically distinct cellular phenotype

**Figure 4:**
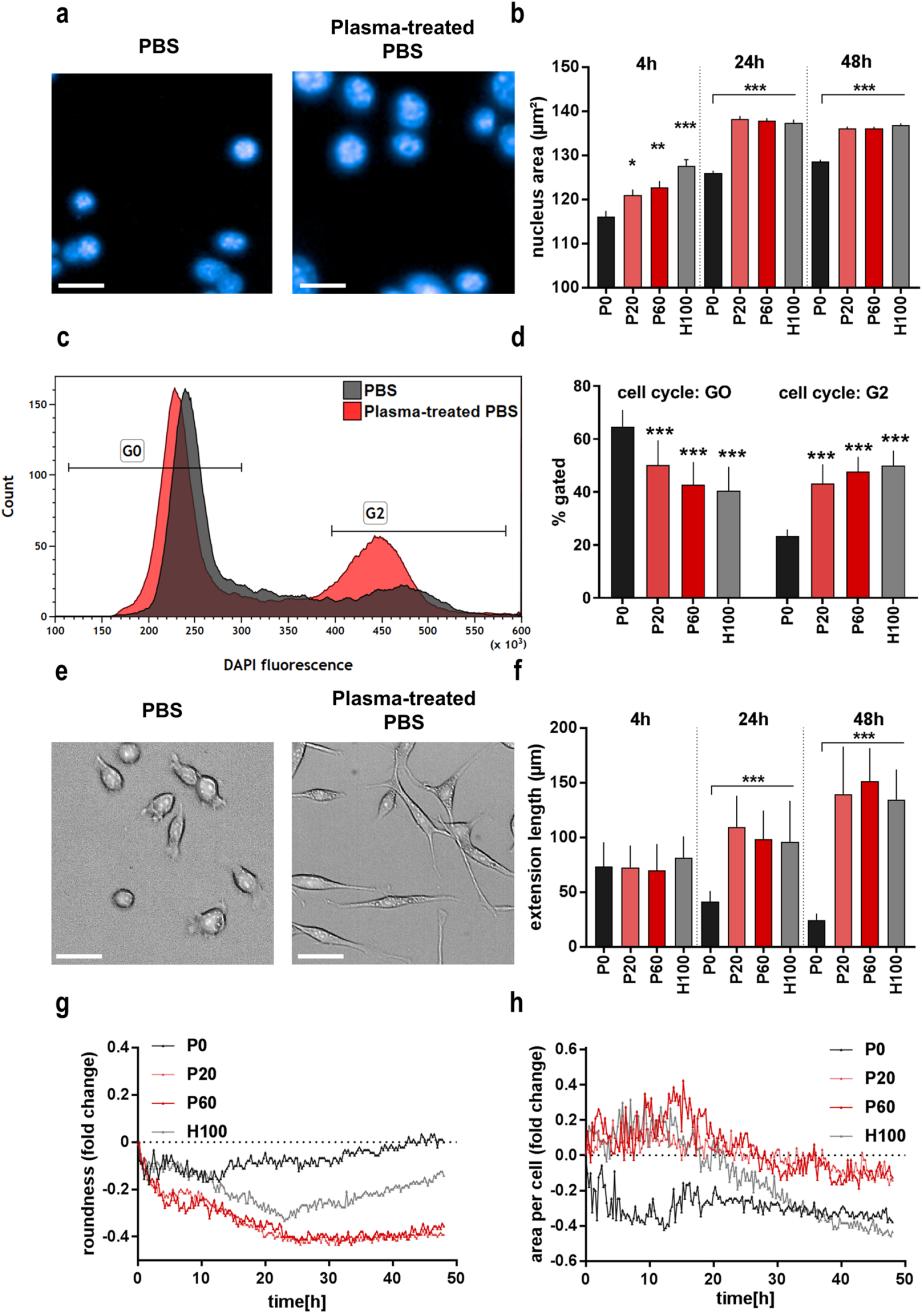
Plasma-treated saline induced a distinct morphological phenotype in CT26 cells. (**a**) Representative images showing DAPI^−^ stained nuclei (scale bar = 50 µm); (**b**) quantification of mean nuclear area at different incubation times following exposure to saline solutions; (**c**) flow cytometry overlay histogram of DAPI-stained DNA 48 h after treatment; (**d**) quantitative analysis showing percent of cells in G0 and G2 cell cycle-phase 48 h after exposure to saline solutions; (**e**) representative images showing live CT26 cells in brightfield at 24 h (scale bar = 100 µm); (**f**) image quantification of spindle-like extension analysis at different incubation times following exposure to saline solutions; (**g**) kinetic of mean roundness and (**h**) mean cell area per condition normalized to t = 0 of each condition, imaging data are from of nine fields of view in 4 replicates per condition. Data are presented as mean and SEM of three independent experiments. Data shown for cells are directly after 30 min of exposure to plasma solutions; statistical analysis was carried out with ANOVA; P0 = control PBS, P20 and P60 = plasma-treated PBS, H100 = concentration-matched hydrogen peroxide to P60.

**Figure 5:**
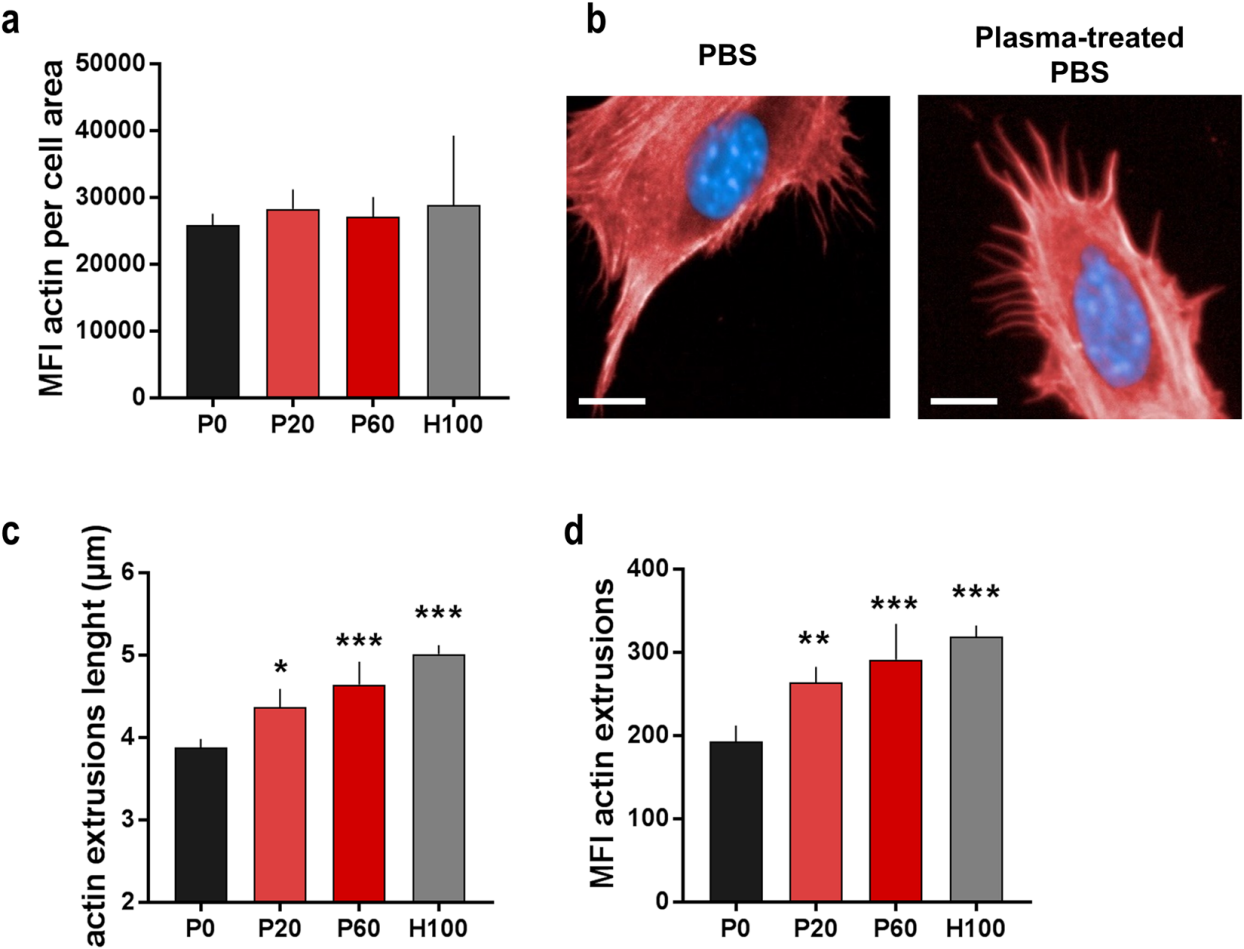
Plasma-treated saline reorganized actin-cytoskeleton. (**a**) Image quantification total phalloidin (staining actin) fluorescence; (**b**) representative confocal images of fixed CT26 cells stained against actin and DNA showing distinct pericellular patterns (scale bar = 10 µm); (**c**) quantification of mean cell extrusion length and (**d**) mean fluorescence intensity (MFI) of extrusions; all data are 4 h after exposure to saline solutions with at least 2000 individual cells analyzed per condition. Data are presented as mean and SEM of one representative of three independent experiments; statistical analysis was carried out with ANOVA; P0 = control PBS, P20 and P60 = plasma-treated PBS, H100 = concentration-matched hydrogen peroxide to P60.

Having identified the cytotoxic effects of our treatment, the next question was whether the saline solutions affected the phenotype of CT26 cells. Exposure to plasma-treated or H_2_O_2_-supplemented saline altered the cells’ nuclear morphology (Fig. 4a). In particular, the mean nuclear area was significantly enhanced from 4 h up to 48 h after treatment (Fig. 4b). Nuclear swelling was similar in both the plasma and peroxide group. Flow cytometry of DNA content showed that in both groups the proportion of cells containing twice the amount of chromatin was significantly increased (Fig. 4c,d). These results argued for a cell cycle arrest in CT26 cells following incubation with oxidizing saline solutions, and the findings were confirmed in MC38, PDA6606, and HaCat cells (Supplementary Fig. S3). It should be mentioned that cell cycle arrest responses in response to toxic stimuli are not unique to malignant cells, as seen with side-effects of many chemotherapeutics in cancer patients on e.g. hair growth and hematopoiesis. We next thought to utilize high content image quantification to discern effects of oxidation on life cell morphology. Similar to cell culture medium controls without exposure to PBS (data not shown), PBS-treated cells had rather round cell bodies with few bipolar cells (Fig. 4e). By contrast, cells exposed to oxidants showed distinctly elongated and spindle-shaped cell bodies with partially branched extensions. The mean length of these extensions was significantly increased in cells 24 h and 48 h after exposure to oxidizing liquids **(**Fig. 4f**)**. Hourly assessment of mean cellular roundness (normalized to mean roundness of each condition at t = 0) was performed over two days to identify the kinetic of morphological alterations (Fig. 4g). Initially, the roundness of cells decreased gradually regardless of the treatment condition. At 12 h, control cells (P0) reversed this tendency, regaining initial roundness at 48 h. At 24 h, H_2_O_2_ PBS treated cells (H100) reversed the decline but without reaching initial roundness at the end of observation. For plasma-treated PBS, roundness continuously declined over time and remained similarly low until observation stopped. These results strongly indicated a phenotypical shift in CT26 colon carcinoma cells in response to plasma-treated but not H_2_O_2_-enriched PBS. This was underlined by results from mean individual cell area, where plasma-treated saline initiated larger cells up to 24 h post-treatment and again produced a different kinetic compared to control and H_2_O_2_ PBS (Fig. 4h). The formations of cell extensions, greater area per cell, and decrease in cell roundness was repealed by adding catalase to all treatment groups (Supplementary Fig. S3). The systematic of these changes in cellular phenotype following exposure to oxidants solutions could also be shown in two the tumor cell lines and HaCaT keratinocytes (Supplementary Fig. S3), not suggesting a tumor-specific effects of oxidizing saline solutions to cell morphology. In principle, morphological alterations are often linked to a different profile in actin cytoskeleton distribution. We therefore quantified total phalloidin fluorescence (a stain for actin) directly (data not shown) and 4 h following treatment but did not notice any difference in total actin content between treatment regimens (Fig. 5a). However, spinning-disc confocal microscopy suggested a transformation in actin distribution patterns. Specifically, a difference in small pericellular signatures in cells exposed to oxidizing saline solutions was identified 4 h post treatment (Fig. 5b). The total length (Fig. 5c) as well as actin (Fig. 5d) content of extrusions directed radially away from the cellular body into the surrounding region were significantly increased. However, the functional consequences of these findings were not clear. One possibility was the induction of epithelial-mesenchymal-transition (EMT), a cellular phenotype involved in supporting tumor cell metastasis^36^. As this would be an unfavorable effect of oxidizing solutions for cancer treatment, we next sought to investigate CT26 cellular function and phenotype with regard to EMT.

### Oxidizing saline solutions decreased cell motility and did not induce an EMT phenotype

**Figure 6:**
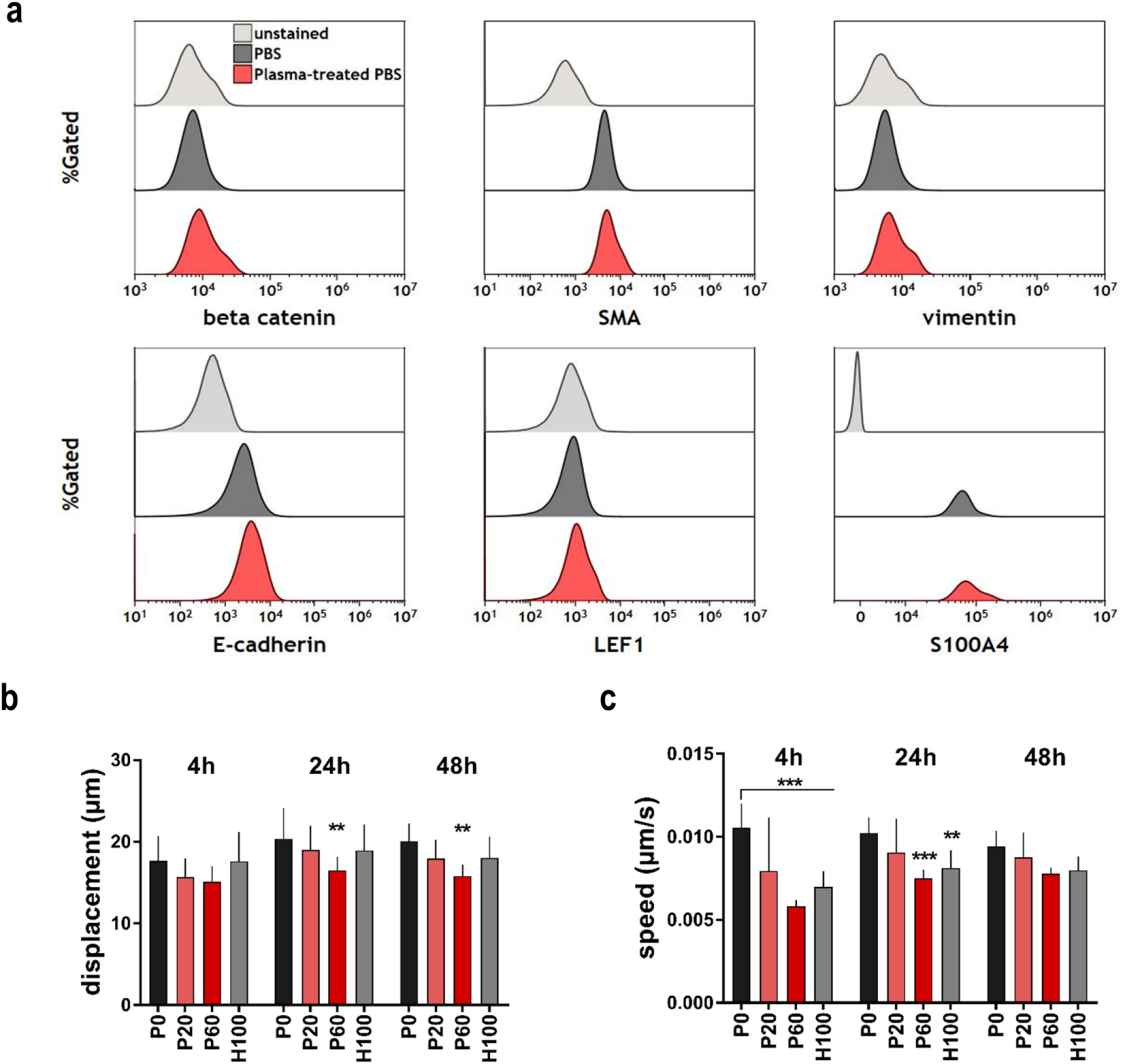
Plasma-treated saline reduced CT26 cell motility and did not promote EMT. (**a**) Representative flow cytometry histograms of CT26 cells stained for the epithelial-mesenchymal-transition (EMT) markers β-catenin, SMA, vimentin, E-cadherin, LEF1, and S100A4, no increase was observed 48 h after exposure to saline solutions (P0 and P60); unstained cells were exposed to plasma-treated saline solution (P60); (**b**) quantification of microscopy time-lapse images of mean cellular displacement and (**c**) speed of viable (PI^−^) cells at 0–4 h, 20–24 h, and 44–48 h following exposure to saline solutions. Data (**b**,**c**) are mean and SEM of three independent experiments; statistical analysis was carried out with ANOVA; P0 = control PBS, P20 and P60 = plasma-treated PBS, H100 = concentration-matched hydrogen peroxide to P60.

Upregulation of β-catenin and the transcription factor LEF1 are key events in EMT^36,37^. Furthermore, upregulation of vimentin, smooth-muscle actin (SMA), and S100A4, or loss of E-cadherin are accompanied with a disparaging mesenchymal tumor phenotype^38^. Using 7-color flow cytometry, expression levels of all mentioned markers were investigated in following exposure to oxidizing saline solutions. Neither an up nor down-regulation of any marker was identified in CT26 colon cancer cells (Fig. 6a). The small “shoulder” on the right of some histograms was equally present in unstained cells (which were exposed to P60 PBS) and stained, P60 treated cells. The shoulder in unstained but P60-treated cells was due to a relative increase of larger cells in these treatment regimens, which gave more auto-fluorescence as seen in mean forward scatter size (data not shown). To understand functional consequences of morphological alterations observed with our treatment, time-lapse video microscopy was utilized to investigate cellular dynamics in viable (PI^−^) cells at different time points post exposure to oxidizing saline solutions. Especially for plasma-treated and partially for H_2_O_2_ saline, total cellular displacement was decreased over 4 h intervals at 4 h, 24 h, and 48 h (Fig. 6b). More strikingly, average cell speed of viable CT26 cells was markedly decreased with all treatments (Fig. 6c). This was most evident 4 h after treatment and with the P60 condition. Interestingly, cellular motility (in tendency) and displacement (significantly) was reduced even 48 h after initial exposure to oxidizing saline solutions. Together with a lack of upregulation of key EMT markers, these results suggest that exposure to the oxidizing solutions does not promote a more motile and thus increasingly metastatic EMT-phenotype of CT26 colon cancer cells, despite profound morphological alterations.

### Exposure to oxidizing saline solutions induces immunogenicity in colon cancer cells

**Figure 7:**
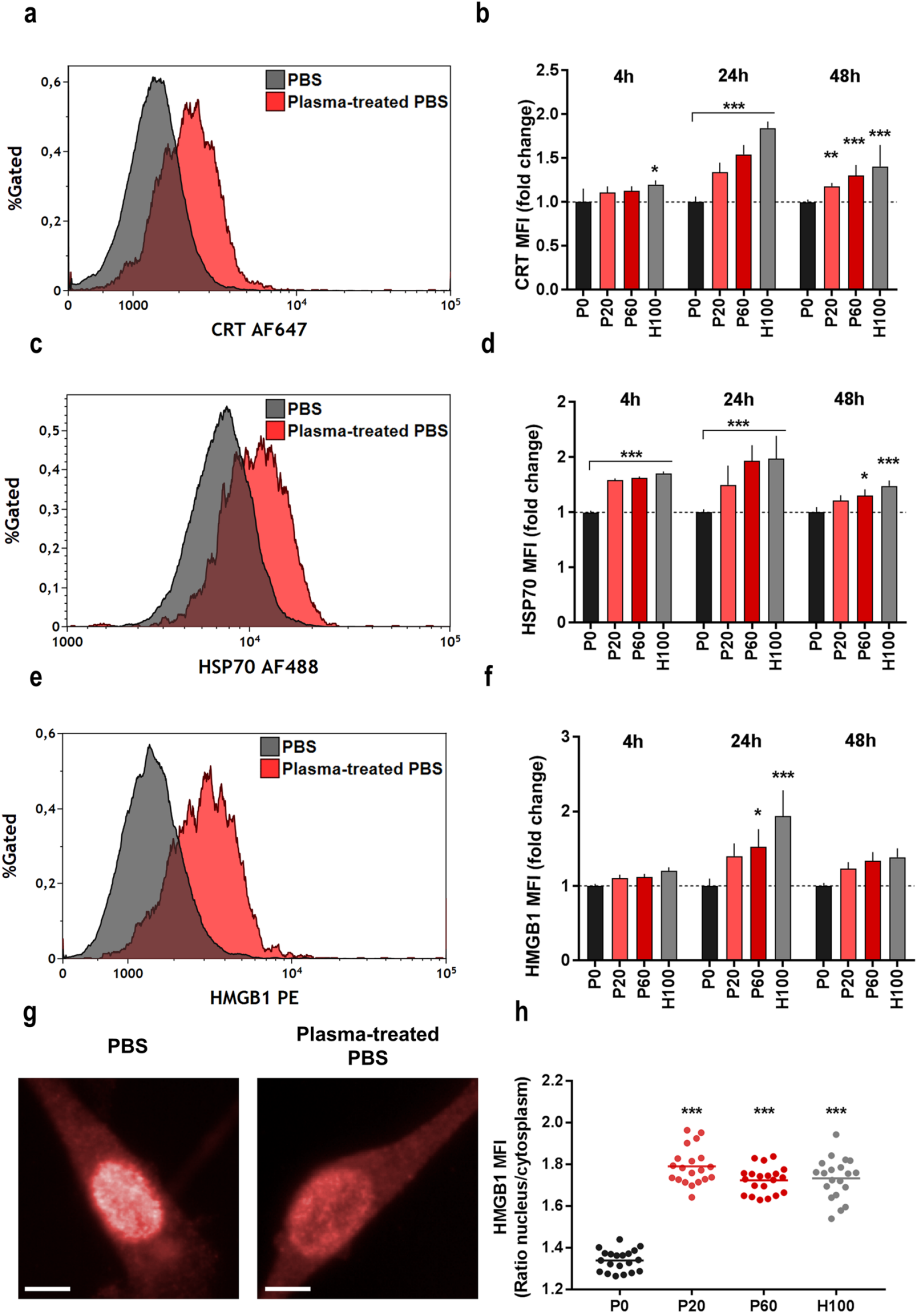
Plasma-treated saline increased translocation and expression of immunogenic cell death (ICD) surface markers in CT26 cells. Representative flow cytometry histograms of CT26 cells stained against the ICD markers calreticulin (CRT; **a**), HSP70 (**c**) and HMGB1 (**e**) to control or plasma-treated PBS and their respective quantification (**b**,**d**,**f**) showed an increased expression of surface markers, especially after 24 h; **(g**) representative DPC and HMGB1 (AF647) images (scale bar = 10 µm); (**f**) ratio of mean fluorescence intensity of HMGB1 in nuclear and cytosolic area 24 h after treatment; imaging data are from of nine fields of view in 4 technical replicates per condition. Data (**b**,**d**,**f**) are mean and SD of three independent experiments; statistical analysis was carried out with ANOVA; P0 = control PBS, P20 and P60 = plasma-treated PBS, H100 = concentration-matched hydrogen peroxide to P60.

Research suggested that not necessarily the extent but rather the mode of cell death can be decisive in antitumor responses, especially with involvement of cells of the immune system^33^. It is known that tumor cells have the ability to evade immune response and therefore decrease the expression of molecules triggering immune activation. Therefore, we thought to determine the release and/or expression of several markers of immunogenic cell death (ICD), which can be sufficient to induce antitumor-immunity. Following exposure to oxidizing saline solutions, we identified an upregulation of key ICD-markers, such as calreticulin (CRT), heat shock protein 70 (HSP70), and high-mobility-group-protein B1 (HMGB1) on the cell surface of CT26 colon-cancer cells (Fig. 7a,c,e) using flow cytometry. The H_2_O_2_-degrading enzyme catalase abolished this increase in all groups (Supplementary Fig. S4). Strikingly, our treatment induced an upregulated of ICD-markers also in MC38 and PDA6606 tumor cells, but not in non-malignant HaCat keratinocytes (Supplementary Fig. S4). For CRT, we confirmed its significantly increased accumulation on PI^−^ (live) CT26 cells exposed to oxidizing saline solutions using high content imaging (Supplementary Fig. S4).In general for CT26 cells, ICD marker expression peaked at 24 h (Fig. 7b,d,f). To confirm a role of HMGB1, quantitative microscopy revealed a significantly increased translocation of the nuclear protein to the cytosol (Fig. 7g) with plasma and H_2_O_2_ treatment conditions (Fig. 7h). In addition, tumor cell-derived HMGB1 was secreted in the cell culture supernatants to a significantly higher extent compared to PBS controls **(**Supplementary Fig. S4). For another DAMP, ATP, we could not detect an increase but a decreased in cells supernatant after 10 min of incubation in oxidizing saline solutions (Supplementary Fig. S4). Not only CRT, heat-shock proteins, and HMGB1 but also cytokines and chemokines are major determinants for efficient antitumor immunity. Parallel quantification of 12 soluble targets in CT26 cell culture supernatants (sampled at 24 h) revealed an increase in tendency for IL2, CXCL9, MCP-1, and IFNγ as well as a significant increase for IL1β, IL6, IL12p70, CCL4, and TNFα with oxidizing saline treatment conditions (Supplementary Fig. S5). At the same time, also levels of two rather anti-inflammatory factors (IL4, and IL10) were elevated with another one (TGFβ, a major immunosuppressive mediator) being decreased. In summary, plasma or H_2_O_2_-treated PBS induced a pro-immunogenic phenotype in several cancer lines concomitant with an overall pro-inflammatory chemokine/cytokine profile.

### Oxidized saline decreased tumor burden *in vivo* and increased macrophage count and T cell activation

**Figure 8:**
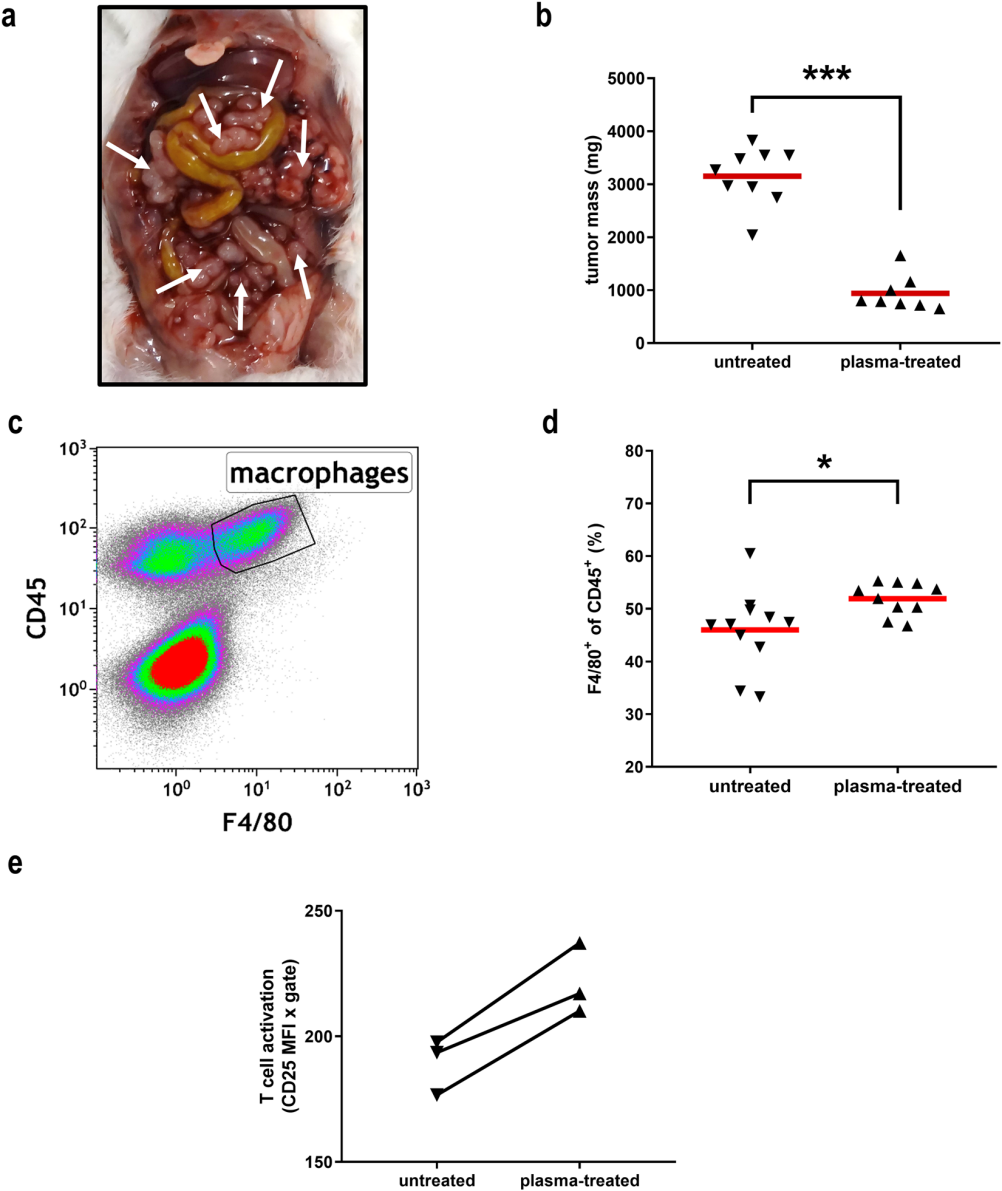
Oxidized saline decreased tumor burden *in vivo* and increased macrophage count and T cell activation. (**a**) Representative image of intraperitoneal burden of CT26 colon cancer cells in Balb/C mice; (**b**) tumor weight in animals receiving saline (untreated) or plasma-treated saline; (**c**) gating of intratumoral macrophages (F4/80^+^ cells of CD45^+^ leukocytes) of digested tumors assessed via flow cytometry; (**d**) quantification of intratumoral macrophages; (**e**) pooled (one sample from two mice of the same group) splenic CD4^+^ T cell activation (CD25 mean fluorescent intensity × % CD25^+^) of animals receiving saline (untreated) or plasma-treated saline after 24 h of incubation with heat-killed CT26 tumor cells *ex vivo* (*p* < 0.018, ratio-paired t test). Data (**b**,**d**) are mean of 8–9 animals, results were statistically compared with t test.

In order to evaluate the effect of plasma-treated saline *in vivo*, mice with CT26 peritoneal carcinomatosis (Fig. 8a) were treated either with untreated or plasma-treated saline. Repetitive application of plasma-treated saline significantly decreased the tumor mass to one third of that of the control group (Fig. 8b). Tumors are often infiltrated with different types of immune cells that together with stroma cells form the tumor microenvironment (TME), which can either spur or halt cancer growth^39^. Macrophages, positive for the surface markers CD45 and F4/80 in murine systems (Fig. 8c), play an important role in the TME, and an increase of these cells was observed in plasma-treated animals (Fig. 8d). These innate immune cells, capable of antigen-presentation to antigen-presentation to adaptive immune cells such as T cells, could have been attracted by dying cancer cells in response to repetitive exposure to plasma-treated saline. To address whether our treatment could have elicited antitumor T cells responses, splenocytes of the control and plasma group were incubated with heat-killed CT26 cells *ex vivo*. Assessment of expression levels of CD25 revealed an increase in CD4^+^ T cells (but not CD8^+^, data not shown) in the plasma over the control group (Fig. 8e). These results suggested that plasma-treated saline was able to decrease tumor burden, increase the number intratumoral macrophages, and promoted the elicitation of antitumor T cell responses targeted against CT26 colorectal cancer cells.

## Discussion

The aim of this study was to investigate a physical plasma-treated phosphate-buffered saline solution in principle suitable for peritoneal lavage of tumor patients with colon cancer metastasis. The solution was not only cytotoxic in the murine cancer cell lines CT26, MC38, and PDA6606 as well as in CT26-bearing mice, but also evoked a pro-immunogenic phenotype in these cells.

Our treatment led to a significant upregulation of calreticulin (CRT) on colon cancer cells. CRT is a leading marker for ICD and a chaperon derived from ER that translocates to the cell surface to serve as *eat-me* signal for phagocytosis of tumor material^40–43^. Recent studies suggested CRT to serve as marker for positive therapeutic outcome in tumor-patients^44–46^. We also observed other damage-associated molecular patterns (DAMPs^32^) such as heat-shock protein 70 (HSP70) and high-mobility-group-protein B1 (HMGB1) to be increased on the tumor cell surface with oxidizing treatment. Surface HSP70 is a chemoattractant for monocytes and neutrophil granulocytes and can lead to maturation of dendritic cells^32^. The nuclear protein HMGB1 can bind to several receptors like TLR2, TLR4, RAGE, and Tim3. These are known for their immunogenic signaling cascades^32^ and induce maturation of dendritic cells (DCs), DC-dependent priming of T-cells^47^, and suppression of regulatory T-cells (Tregs)^48^. Hence, by upregulation of these surface markers, our treatment could have the potential to activate antitumor immunity. This was illustrated by the T cell activation observed in our study, which is in line with previous findings^49,50^ Such notion was further supported by analysis of several immunomodulatory mediators in the supernatant of cancer cells that were exposed to saline solutions. This included increased secretion of interleukin (IL) 1β, IL6, IL12p70, cc-chemokine ligand 4 (CCL4), and tumor necrosis factor α (TNFα). Moreover, IL2, c-x-c motif ligand 9 (CXCL9), monocyte chemotactic protein 1 (MCP-1), and interferon γ (IFNγ) were increased in tendency. Indeed, we observed an increase of intratumoral macrophages in our murine model, which supports the notion of improved 5-year survival observed in patients with colorectal cancer^51^. In general, chemokines/cytokines are potent immune-effectors in cancers^52^, crucial for shaping the tumor microenvironment *in vivo*^39,53,54^. The pyrogenic interleukin 1β is a major inducer of inflammation and macrophage activation, and mediates apoptosis. IL6 enhances proliferation and function of T and B cells^55,56^. IL12p70 is a prominent inducer of anti-tumor immunity mostly through the stimulation of natural killer (NK) cells and cytotoxic T lymphocytes (CTLs) and their subsequent secretion of IFNγ. IL6 diminished tumor growth of colorectal cancer in pre-clinical studies^57,58^ The chemokine CCL4 works as attractant for a wide spectrum of immune cells^59^ whose presence positively correlates with survival in colorectal cancer patients^60–63^. We also found increased levels of TNFα that is an inducer of apoptosis^64^. Immuno-suppressive TGFβ, decreased in our study, suppresses effector T cell function and enhances growth of stromal cells and extracellular matrix components, which in turn can promote unwanted mesenchymal transmission^65–67^. By contrast, increased levels of IL10 are not associated with favorable prognosis, because it can decrease cross-presentation of tumor antigens by dendritic cells to T cells, for example^68,69^. In general, chemokines and cytokines modulate inflammation, which in turn affects tumor cell behavior and phenotype^70^. For another DAMP, adenosine triphosphate (ATP), a decrease was observed in supernatants of CT26 cells exposed to oxidizing saline solutions. However, original studies identifying the immune-stimulating effects of ICD-induced CT26 colorectal cancer cells did not report on ATP but rather CRT being crucial for immuno-protection in mice^41,43^. Hence, despite the lack of the “find-me” signal ATP, we propose that oxidized saline treatment could increase the *in vivo* immunogenicity of three tumor cell lines (but not non-malignant HaCaT keratinocytes) due to a significant increase in the ICD-markers CRT, HMGB1, and HSP70/90^71^.

Exposure to plasma-treated PBS led to phenotypical changes in CT26 cells, which were visible even 48 h after initial contact. The consistently enlarged nuclei and cell area as well as the decelerated cell growth and cell cycle are hallmarks of cellular senescence^72^. Senescence was previously observed in melanoma cells following exposure to cold physical plasma^73^. Interestingly, the authors found cell senescence only for treatment conditions with moderate toxicity, which corroborates the findings of our study. Many tumors have the potential to bypass cell cycle arrest in order to divide and grow dysregulated^74^. Cellular senescence was previously found to be associated with increased intracellular oxidized protein content^75^, an observation also made after plasma treatment^76^. Moreover, we found changes in actin distribution in CT26 cells. It is known that ROS act as signaling molecules for several downstream pathways that lead to remodeling of the cytoskeleton in seconds to minutes^77,78^. The unwanted mesenchymal tumor phenotype shows increased migration, proliferation, and expression of molecules responsible for rapid cytoskeletal rearrangement^79^ allowing them to dispatch from primary tumors^36^. Our results with plasma-treated PBS contradict these characteristics. We found a sustained weakened cell growth, decreased cell motility, and a lack of increased expression of important EMT markers. Recent studies suggested senescence and its phenotypic alterations to repress malign, epithelial tumor phenotypes^80^.

For the *in vitro* effects of plasma jets in biological systems, H_2_O_2_ was suggested to be an important molecule^81^. For this reason, we mimicked its lone effect by using a plasma treatment concentration-matched amount of H_2_O_2_ as positive control. The plasma gas phase is rich in hundreds of different reactive species^82^ but in liquids they quickly decompose to a few stable ones^83^. Superoxide, for example, is abundant^84^ but rapidly dismutates to H_2_O_2_ in solutions. Therefore, plasma-derived superoxide identified in our work unlikely contributed to biological effects directly. Along similar lines, nitric oxide and related reactive nitrogen species yield nitrite that further reacts to nitrate in plasma-treated liquids^85^. Although both are of low reactivity, there is a hypothetical framework attributing them a role in specifically mediating plasma-derived antitumor effects^86^. The assumption is that tumor cell membrane-resident NAPDH oxidases (NOX) may generate cell-derived superoxide, which may react with nitric oxide to cytotoxic peroxynitrite; the latter, however, may also derive from H_2_O_2_ and nitrite (both identified in plasma-treated PBS in this study)^87^. In our assays, immediate oxidation of cells was significantly enhanced in plasma-treated compared to H_2_O_2_ saline, despite identical concentrations of H_2_O_2_ (but absence of hypochlorous acid)^88^. This points to H_2_O_2_-independent reactions or additional/unknown reaction intermediates^89^. More evidence of the importance of this chemistry was provided by pharmacological inhibition of NOX that strongly attenuated the effect of plasma on cancer cells^90^. We have previously seen the inability of a (to the plasma treatment time concentration-matched) amount of H_2_O_2_ to replicate functional consequences in human neutrophils seen after direct plasma treatment^91^. Of note, neutrophils carry large numbers of NOX molecules on their membranes^92^. In the present work, we found rather small differences between plasma-treated and H_2_O_2_-supplemented phosphate-buffered saline. As major exception, killing of colorectal cancer cells in 3D tumor spheroids, a more realistic model in oncological research^93^, performed significantly better with plasma-treated PBS compared to H_2_O_2._

Tumor-bearing mice benefited from direct exposure of bulk tumors to the short-lived reactive species generated by cold plasmas^94–96^. Recently, several groups suggested to expose liquids to plasmas for tumor therapy. We previously observed toxic effects of such liquids *in vitro* and *in vivo*, including diminished cell growth, proliferation, and metabolic activity as well as induction of apoptosis but not necrosis in a model of pancreatic cancer^24^, which supports earlier work^97–99^. We could confirm this findings and translate it to another tumor model using plasma-treated saline. However, many studies used plasma-treated cell culture medium to inactivate cancer cells^100–102^. While this is an approach proven to be effective also in tumor-bearing mice^24,25,100^, the clinical translation of this concept may be limited.

Double-distilled plasma-treated water has been shown to be effective against cancer cells^103,104^ but its use is discouraged in the clinical setting due to unfavorable osmolality. Sodium chloride (0.9%) solution is a clinically accepted solution, and phosphate-buffered saline (PBS) is very similar to that. Even following three weeks of storage we did not find any loss of the major oxidant H_2_O_2_ deposited by this argon plasma jet into solution^89^. Previous studies on plasma-treated medium stored up to 7 days showed a drastic H_2_O_2_ loss over time^105,106^, presumably due to reaction with protein thiols^107^. Yet, the immediate H_2_O_2_ concentration of a plasma jet-treated liquid is independent from its protein content^108^, suggesting rather slow reaction kinetics for decay. Therefore, plasma-treated saline solutions that can be frozen and stored for longer periods of time should further be tested for its use as adjuvant for intraperitoneal cancer treatment *in vivo* experiments and potentially also regarding its clinical applicability.

Our study has limitations. First, we did not investigate the molecular machinery responsible for immunogenic cancer cell death observed. Especially caspase 8 and other signaling events upstream of the ER-stress sensor PERK may be important for the effects found in our study^109^. Second, a number of positive controls on the events of e.g. apoptosis, ROS generation, and translocation of ICD-relevant molecules may further context the presented findings within the vast body of literature available on CT26 cell death^110–112^. Third, also other liquids in clinical use may have the potential to elicit the effects observed in this study, e.g. Ringer-Lactate solutions studied previously with regard to plasma treatment^97^. Fourth, despite our willingness to perform the animal experiments including an H_2_O_2_ control group, the local ethics committee denied this request and only approved the two groups presented. Notwithstanding, the here presented findings pose a promising and safe new therapeutic avenue for the treatment of colorectal cancer that warrants further investigations.

## Materials and Methods

### Cell culture

Murine colon carcinoma cells (CT26 and MC38) as well as the murine pancreatic carcinoma cells (PDA6606) and human non-malignant keratinocytes (HaCat) were maintained in Roswell Park Memorial Medium (RPMI) 1640 (Pan Biotech), containing 10% fetal bovine serum, 2% glutamine, and 1% penicillin-streptomycin (all Sigma). Cells were incubated at 37 °C, 95% humidity, and 5% CO_2_. Sub-culturing was performed twice a week. For experiments, 1 × 10^4^ cells were seeded in flat-bottom 96-well plates (Eppendorf). These plates have a rim, which can be filled with sterile water to prevent excessive evaporation during incubation and subsequent alterations in the outer wells of the plate (edge effects).

### Plasma and treatment

To produce a sufficient amount of treatment solution, 50 ml of phosphate-buffered saline solution (PBS) without Ca^2+^ and Mg^2+^ (PAN Biotech) was exposed to cold physical plasma in a 150 ml glass beaker for 20 or 60 minutes. The atmospheric pressure argon plasma jet kINPen^113^ (neoplas tools) was used working with five standard liters of argon gas (99.9999% purity; Air Liquid) per minute. The physical characterization of this plasma jet were thoroughly investigated in previous studies and are summarized in^114^. After plasma treatment, a predetermined amount of double-distilled water was added to PBS to compensate for evaporation effects that otherwise would affect the osmotic pressure of the solution on cells. Thereafter, the PBS was stored in aliquots at −20 °C for up to three weeks. The stability of hydrogen peroxide (H_2_O_2_, a main component of the plasma treatment solutions) over that time is shown in Fig. 1b. Before utilizing the PBS for experiments, it was freshly thawed in a water bath at 37 °C. After use, it was discarded. No repeated freeze-thaw cycles were used for cell experiments. Untreated PBS worked as negative control, 100 µM H_2_O_2_ (Sigma) as positive control, and were handled in the same manner. The 100 µM of H_2_O_2_ match the concentration of H_2_O_2_ deposited with the plasma jet in PBS after treatment of 50 ml for 60 min. For treatment of cells, cell culture medium was discarded and 100 µl of PBS was added to each well for 30 min (except for initial kinetic studies, see Fig. 1c). After 30 min, PBS was replaced with fully supplemented cell culture medium, and downstream experiments were performed either immediately or after 4 h, 24 h, or 48 h of further incubation. To scavenge H_2_O_2_ as additional control regimen, the enzyme catalase was added in a concentration of 20 µg/ml to plasma-treated or H_2_O_2_-containing PBS (referred to as oxidizing saline solutions) immediately after their generation and prior to freezing. These control solutions were also stored at −20 °C and were used in the same way as PBS solutions without catalase. The motivation of testing frozen PBS is to mimic a clinical situation, in which a plasma-treated liquid may be needed onsite immediately. This would leave no time to generate and, importantly, sterile filter the solution prior to application. Hence, a possibility would be to generate plasma-treated liquids at a dedicated facility, sterile filter this solution into medical bags, and store it at −20 °C close to the operating theater to make it available immediately if needed (after heating, as done with other medical liquids already today).

### Oxidant detection

H_2_O_2_ in PBS was quantified using the *Amplex UltraRed* reagent (Thermo Fisher) according to the manufacturer’s instructions. Fluorescence was determined using a multiplate reader (Tecan) at λ_ex_ 530 nm and λ_em_ 590 nm. For detection of intracellular reactions involving reactive species and catalytic enzymes, cells were incubated with chloromethyl 2′,7′-dichlorodihydrofluorescein diacetate (CM-H_2_DCF-DA; Sigma) before treatment. The dye diffuses into cells and fluoresces upon reaction with reactive species in the presence of intracellular oxidases. Live cells were imaged with a high content imaging system (Operetta CLS; Perkin Elmer) with a 20x objective (NA 0.4) in brightfield (BF), digital phase contrast (DPC), and fluorescence (λ_ex_ 475 nm and λ_em_ 505 nm) channel. Images were analyzed with Harmony high content imaging and analysis software 4.8 (Perkin Elmer). Cells were segmented via DPC, and mean fluorescence intensity (MFI) per cell and field of view were calculated. At least 2000 cells were used for analysis of each condition. Nitrate and nitrite were quantified using the *Nitrate/Nitrite Colorimetric Assay Kit (Griess Assay)* according to the manufacturer’s instruction (Cayman Chemical) directly after plasma treatment of saline as well as after freezing and one week later thawing these solutions. In order to quantify the deposition of superoxide in freshly treated saline as described before^108^, the solutions were incubated with 1 mg/ml oxidized cytochrome c (Sigma) and 20 µg/ml catalase (Sigma). The absorption was measured directly after treatment at 590 nm utilizing a multiplate reader (Tecan).

### Metabolic activity

Immediately, 20 h, or 44 h after exchange of PBS with cell culture medium, cells were incubated with 100 µM 7-hydroxy-3H-phenoxazin-3-on-10-oxid (resazurin; Alfa Aesar) for 4 h. Resazurin can be transformed to highly fluorescent resorufin via redox reductions indicating metabolic activity in cells^115^. Fluorescence increase was quantified using a multiplate reader (Tecan F200) at λ_ex_ 530 nm and λ_em_ 590 nm and normalized to samples that had received untreated PBS.

### Time lapse imaging and morphological analysis

For time lapse imaging experiments, propidium iodide (PI; Sigma) was added to detect terminally dead cells. The cell culture plate was incubated in the high content imaging system at 37 °C and 5% CO_2_ for 48 hours. Several images per hour were taken using an air 20x objective (NA = 0.4) in the BF, DPC, and fluorescence (λ_ex_ 550 nm and λ_em_ 630 nm) channel. The rim of the 96-well plate was filled with sterile water to protect cells from excessive evaporation. The imaging system is equipped with a laser-based autofocus to maximize image focus for subsequent software segmentation of cells and their properties using Harmony software. Alternatively, the cells were fixed and imaged at several time points. To determine mean nuclear area after treatment, cells were fixed with 70% ethanol, washed, and incubated with 4′,6-diamidin-2-phenylindol (DAPI; BioLegend) before imaging and analysis with the high content system using the above mentioned conditions (except for the fluorescence setup, which was λ_ex_ 365 nm and λ_em_ 405 nm). For analysis of actin distribution, cells were cultured and treated in glass-bottom 96-well plates (Cellvis) and exposed to PBS as described. For imaging experiments, cells were fixed and incubated with flash phalloidin NIR 647 (BioLegend). For quantification of total actin levels, imaging conditions were as stated above (except for the fluorescence setup, which was λ_ex_ 630 nm and λ_em_ 660 nm). For this, cells were segmented using actin fluorescence, and the MFI for each cell per well was quantified. To resolve and quantify actin extrusions at the cell membrane, a 40x water immersion objective (NA = 1.1) was utilized for acquisition of several true-point confocal image stacks per field of view. To determine the nuclear and cytosolic distribution of high-mobility-group-protein B1 (HMGB1), cells were fixed with paraformaldehyde (PFA) and exposed to permeabilization buffer (both BioLegend) before overnight incubation with an unconjugated anti-HMGB1 antibody (Abcam). The next day, a secondary antibody conjugated with Alexa Fluor (AF) 647 Plus (Thermo Scientific) was added, and imaging was carried out following the procedures described above (except for the fluorescence setup, which was λ_ex_ 650 nm and λ_em_ 665 nm). To image calreticulin (CRT), an unconjugated CRT antibody (Abcam) was added for 1 h before labeling with a secondary AF647 Plus antibody (Thermo Scientific). PI was used to exclude dead cells for analysis. The high content imaging system acquires images with a sCMOS camera (4.7MP) at 16 bit; binning was 1. High performance 30-core computers were utilized for data analysis of about 200,000 single images in total across all imaging experiments performed in this study.

### Flow cytometry

To determine apoptosis, cells were detached with accutase, washed with annexin V binding buffer (AVBB), and incubated with annexin V-labeled phycoerythrin (PE) or Caspase 3/7 detection reagent and DAPI (all BioLegend; Caspase 3/7 from Thermo Scientific). For annexin V staining, cells were subsequently washed and suspended in AVBB. Flow cytometry was performed (CytoFlex; Beckman-Coulter). To determine the mean fluorescence intensity (MFI) of Ki-67, a marker for actively dividing cells, celss were detached with accutase, fixed and permeabilized in ice-cold methanol, and stained with anti-Ki-67 PE antibodies (BioLegend). After washing, cells were analyzed by flow cytometry. In addition to untreated PBS control samples, cells from cell culture flasks were used as additional controls to delineate effects of PBS alone on cell proliferation. Using a similar staining procedure as for Ki-67, cells were stained with anti β-catenin antibodies conjugated with Alexa Fluor 488 (AF488; BectonDickinson), anti-Lymphoid Enhancer Binding Factor 1 antibodies conjugated with AF647 (LEF1; Cell Signaling), anti-vimentin antibodies conjugated with PerCP-Cy5.5 (BioLegend), anti-alpha smooth muscle actin (SMA) antibodies conjugated with AF594 (Abcam), anti E-cadherin (E-Cad) antibodies conjugated with PE/Cy7 (BioLegend), and anti-S100A4 antibodies conjugated with AF700 (Novus Biologicals). To determine the expression of proteins involved in immunogenic cell death, we followed the same protocol except the fixation and permeabilization steps. Cells were labeled with antibodies targeting calreticulin AF647 (CRT; Novus), heat shock protein 70 AF488 (HSP70; Abcam), heat shock protein 90 AF700 (HSP90; Novus Biologicals), high-mobility-group-protein B1 PE (HMGB1; BioLegend), and DAPI. Only live cells with intact membranes (DAPI^−^) were used for quantification of these markers via each MFI. Data analysis was performed using Kaluza analysis software 2.1 (Beckman-Coulter). For analysis of tumor lysates from animal experiments, cells were fixed with 4% PFA and stained with antibodies targeted against F4/80 conjugated with PE-Dazzle (BioLegend) and CD45 conjugated with AF700 (BioLegend) as well as DAPI. To assess T cell activation among splenocytes harvested from mice of the control and plasma group, 10^6^ splenocytes (each sample was pooled from two animals of the same group sacrificed at the same time) were co-cultured with 10^5^ heat-killed CT26 cells. After 24 h of incubation, cells were harvested and stained with conjugated antibodies targeted against CD3 (AF700), CD4 (PE-Cy7), CD8 (BV510), and CD25 (APC) to discriminate T cells and IAIE, CD45.R, F4/80, Ly6C (all APC-Cy7), and Zombie NIR (all BioLegend) to discriminate dead and non-T cells. T cell activation was assessed by quantifying the percentage of CD25^+^ T cells and multiplication with the MFI of CD25^+^ T cells in all CD4^+^ T cells, respectively (MFI x gate). The assay was repeated three times with pooled samples from two mice per sample and group.

### Tumor spheroids

Three thousand CT26 cells were seeded per well of a 96-well spheroid cell culture plate (PerkinElmer) that prevents the attachment of cells to support spheroid formation. Plates were centrifuged at 1000xG for 10 minutes and incubated for 72 h. Subsequently, culture medium was replaced with saline solutions. After 30 min, fully supplemented cell culture medium containing sytox green (life technologies) was added as nutrient source and to detect terminally dead cells via microscopy. Hourly over 12 hours, images were acquired using the high content imaging device with environmental control (37 °C/5% CO_2_), a 5x air objective (NA = 0.16), nine z-stack per spheroid ranging from −70 µm to 70 µm, and for each well and fluorescence channel. Filter setup was as described as above for CM-H_2_DCF-DA experiments. For analysis, z-stacks of each well were merged in *Harmony* software using maximum intensity projection. Spheroids were segmented to distinguish them from background, and sytox green mean fluorescence intensity (MFI) within that area was quantified and normalized to t = 0 of each individual spheroid and well. Immediately after imaging was finished, spheroids were collected, digested with accutase, washed, and subjected to flow cytometry to very imaging results.

### Supernatant analysis

Analysis of soluble analytes in cell-culture supernatants was carried out using the Legendplex (BioLegend) multi-analyte assay, which is a bead-based sandwich immunoassay assayed with flow cytometry. The experimental procedure was performed following the suppliers’ instruction. The panel consisted of the following fluorescence encoded beads directed against: interleukin (IL) 1β, IL2, IL4, IL6, IL10, IL12p70, chemokine (C-X-C motif) ligand (CXCL) 9, monocyte chemoattractant protein (MCP) 1, CC-chemokine ligand (CCL) 4, interferon (IFN) γ, tumor necrosis factor (TNF) α, and tumor growth factor (TGF) β. For quantification of the flow cytometry results, specialized data analysis software (Vigene Tech) was utilized. For each analyte, a separate standard curve was calculated, using fifth degree polynomials, with attention to each analyte’s specific detection limits. To quantify HMGB1 in cell culture supernatant, we utilized an enzyme-linked immuno absorbance assay (ELISA; Hoelzel) following the manufacturer’s protocol. ATP content in PBS 10 min after addition of control and oxidizing saline solutions to cells was quantified following the supplier’s manual using an *ATP Detection Assay Kit* (Abcam). Luminescence was then measured utilizing a multiplate reader (Tecan).

### Animal experiments

Mouse maintenance and experimental procedure were thoroughly reviewed and received approval by the State agency for agriculture, food safety, fishery Mecklenburg-Vorpommern (LALLF-MV, application number 7221.3-1-048/18). All animal procedures were performed in accordance with the relevant guidelines and all efforts were made to minimize suffering. Two million CT26 colorectal cancer cells were injected intraperitoneally (i.p.) to initiate disseminated tumor growth. Starting at day two, mice received at total of five i.p. injections of 300 µl of untreated or plasma-treated 0.9% sodium chloride solution (60 min of treatment kINPen treatment of 50 ml) every other day. Tumor nodules were carefully explanted and weighted prior to digestion with a commercially available tumor dissociation kit (GentleMACS; Miltenyi BioTec, Germany). A similar kit was used to make single cell suspensions of explanted spleens.

### Statistical analysis

All experiments were repeated independently several times and the number of repetitions are given in figure legends. Data shown from several experiments are mean and standard error if not indicated otherwise. To compare two different groups, Wilcoxon rank test was applied. For multiple comparison across several groups, one way analysis of variances (ANOVA) was used with Dunnett post testing to untreated control PBS if not stated otherwise. For multiple comparison across several groups and a second parameter (for instance, time), two way ANOVA was used with *Tukey* post testing if not stated otherwise. Asterisks indicate the level of significance as follows: *, **, or *** for the *p*-values < 0.05, < 0.01, or < 0.001. Graphing and statistical analysis was carried out using *prism 7.05* (Graphpad software, USA).

## Conclusion

Plasma-treated and H_2_O_2_-supplemented saline not only induced growth retardation and apoptotic tumor cell death, but also altered the cells’ phenotype in disadvantage to metastatic spread. Plasma treatment was superior to H_2_O_2_ treatment in inducing several morphological alterations and in killing cancer cells grown in 3D tumor-spheroids. In several tumor cell lines, major mediators of anticancer immunity were identified using our treatment regimens, all above calreticulin. Importantly, plasma-treated saline was effectively reducing tumor burden *in vivo* with a potential role of increased immunogenicity as shown by increased amount in macrophages and T cell activation. These results suggest that such a solution should be tested further as possibly promising adjuvant in colorectal cancer therapy to tackle peritoneal carcinomatosis.

## Supplementary information

Supplementary Info

## Data Availability

The datasets generated and analyzed during the current study are available from the corresponding author on reasonable request.
