# Supplementary material for: Physical plasma-treated saline promotes an immunogenic phenotype in CT26 colon cancer cells in vitro and in vivo
Source: Sci Rep. 2019 Jan 24;9:634. doi: 10.1038/s41598-018-37169-3 (PMC6345938; doi:10.1038/s41598-018-37169-3)

**Physical plasma-treated saline promotes an immunogenic phenotype in CT26 colon cancer cells in vitro and in vivo**

Eric Freund^1,2^, Kim Rouven Liedtke^2^, Julia van der Linde^2^, Hans-Robert Metelmann^3^, Claus-Dieter Heidecke^2^, Lars-Ivo Partecke^2^, Sander Bekeschus^1^

1 Leibniz-Institute for Plasma Science and Technology (INP Greifswald), ZIK *plasmatis*, Felix‑Hausdorff‑Str. 2, 17489 Greifswald, Germany

2 Department of General, Visceral, Thoracic and Vascular Surgery, Greifswald University Medical Center, Ferdinand-Sauerbruch-Str., 17475 Greifswald, Germany

3 Oral and Maxillofacial Surgery/Plastic Surgery, Greifswald University Medical Center, Ferdinand-Sauerbruch-Str., 17475 Greifswald, Germany

correspondence to: Sander Bekeschus, Ph.D.

Mail: [sander.bekeschus@inp-greifswald.de](mailto:sander.bekeschus@inp-greifswald.de)

Phone: +49 3834 554 3948

Short title: plasma-treated saline promotes immunogenicity in colon cancer

**Supplementary figure S1:** Plasma treatment generated nitrates, nitrites and superoxide in saline solution.

(**a**) *Griess* assay with standards and samples of treated and untreated phosphate-buffered saline solution (PBS) for the detection of nitrite and nitrate in freshly plasma-treated PBS (**b**) and plasma-treated PBS after freezing and thawing (**c**); measured absorbance for superoxide detection in 2ml of PBS after 150s of plasma treatment. Data are representative out of three independent experiments and show mean and SD; P0 = control PBS, P20 and P60 = plasma-treated PBS, H100 = concentration-matched hydrogen peroxide to P60.

**Supplementary figure S2:** Plasma-treated saline reduced metabolic activity and cell count of different cancer cell lines.

(**a**) metabolic activity of CT26 cells 24h after exposure to oxidizing saline solutions with or without catalase, (**b**) metabolic activity MC38 murine colon cancer cells, PDA6606 murine pancreatic cancer cells, and HaCat human non-malignant keratinocytes after exposure to oxidizing saline solutions; (**c**) image quantification of cell count in all three cell lines of (**b**). Data were normalized on each P0 and show mean and SD of three independent experiments; statistical analysis was carried out with ANOVA; P0 = control PBS, P20 and P60 = plasma-treated PBS, H100 = concentration-matched hydrogen peroxide to P60.

**Supplementary figure S3:** Plasma-treated saline leads to cell cycle arrest and morphological alterations in different cancer cell lines.

(**a-c**) Quantitative analysis showing percent of MC38, PDA6606, and HaCat cells in S, G0, and G2 cell cycle phase; catalase reduced changes in extension length (**d**), area per cell (**e**) and roundness (**f**) in CT26 cells exposed to oxidizing saline solutions; (**g**) representative DPC images of cells 24h after exposure to untreated and plasma-saline and the quantification of the area per cell (**h**) and cell roundness (**i**); imaging data are from nine field of views in 4 technical replicates per condition (scale bar = 20µm). Data are presented as mean (SEM) (d, e, f) of three independent experiments; statistical analysis was carried out with ANOVA; P0 = control PBS, P20 and P60 = plasma-treated PBS, H100 = concentration-matched hydrogen peroxide to P60. Data (**e**, **f, h, i**) were normalized to each P0.

**Supplementary figure S4:** Plasma-treated saline increased translocation and expression of immunogenic cell death (ICD) surface markers in different cancer cell lines.

(**a**) expression of immunogenic surface markers in CT26 cells 24h after exposure to plasma and control saline with or without catalase; (**b**) representative brightfield and anti-calreticulin images yielded with an antibodies conjugated to alexa fluor 647 (scale bar = 10µm) and (**c**) the quantification of CRT mean fluorescence intensity (MFI) in the membrane region; (**d**) secretion of HMGB1 into supernatant was quantified utilizing an enzyme-linked immuno absorbance assay (ELISA); (**e**) ATP release directly after exposure to saline solutions was analyzed via a chemo luminescence kit; quantification of flow cytometry experiments for the expression of the immunogenic surface markers CRT (**f**), HSP90 (**g**), and HMGB1 (**h**) in the cell lines MC38, PDA6606, and HaCat. Data are presented as mean and SEM of three independent experiments; statistical analysis was carried out with ANOVA or t-test (c); P0 = control PBS, P20 and P60 = plasma-treated PBS, H100 = concentration-matched hydrogen peroxide to P60. Data (except d) were normalized to each P0.

**Supplementary figure S5:** Plasma-treated saline induced secretion of immunogenic cytokines and chemokines in colon-cancer cells.

Quantification (in pg/ml) of IL1β, IL2, IL4, IL6, IL10, IL12p70, CXCL9, MCP-1, CCL4 (MIP1β), IFNγ, TNFα, and TGFβ in cell-culture supernatant at 24h following exposure to untreated, plasma-treated, or H_2_O_2_-treated saline as measured via multiplex bead-based immunoassay. Data are mean and SD of 4 technical replicates pooled out of 9 biological replicates; statistical analysis was carried out with ANOVA; P0 = control PBS, P20 and P60 = plasma-treated PBS, H100 = concentration-matched hydrogen peroxide to P60. LOD = limit of detection.


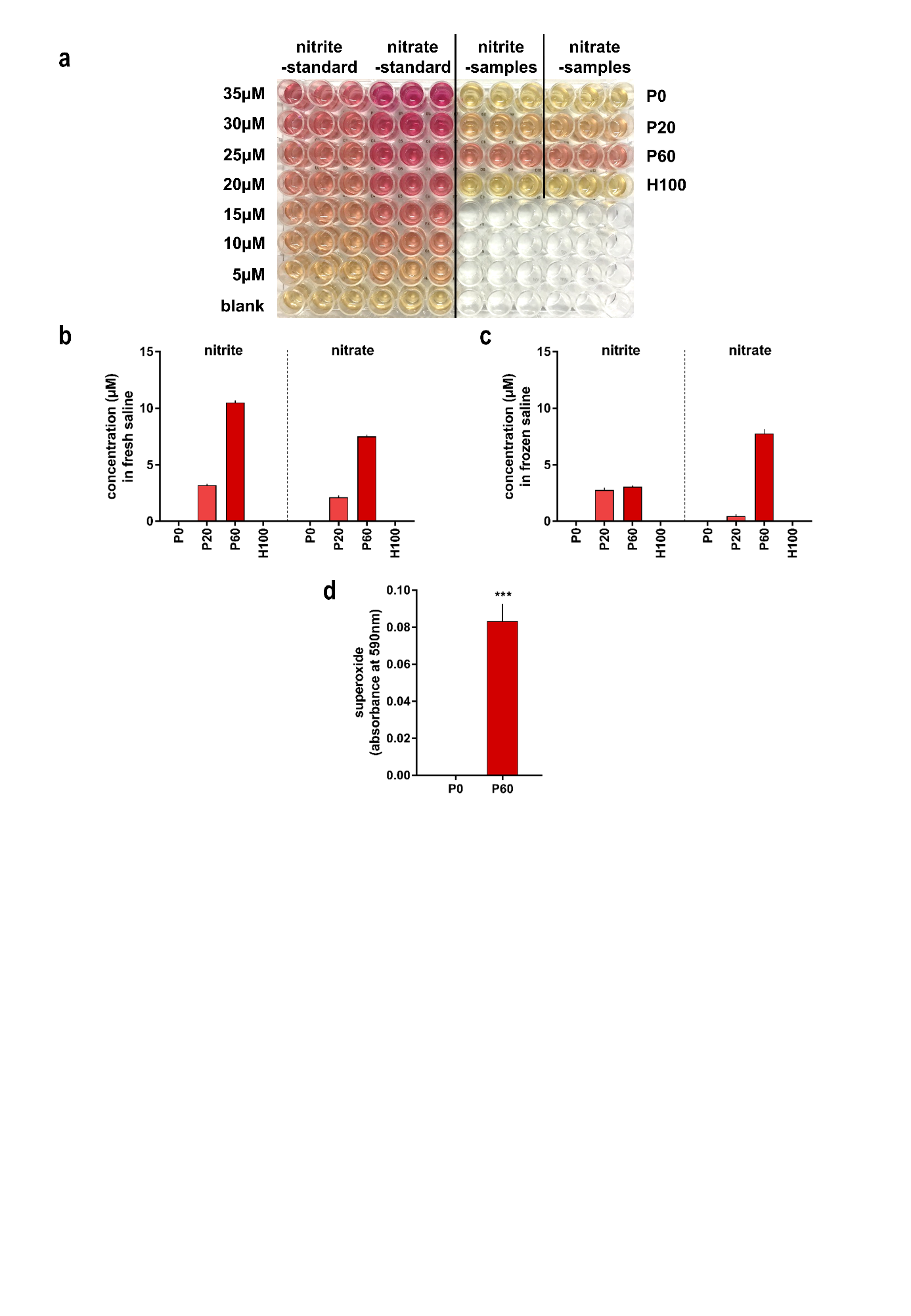


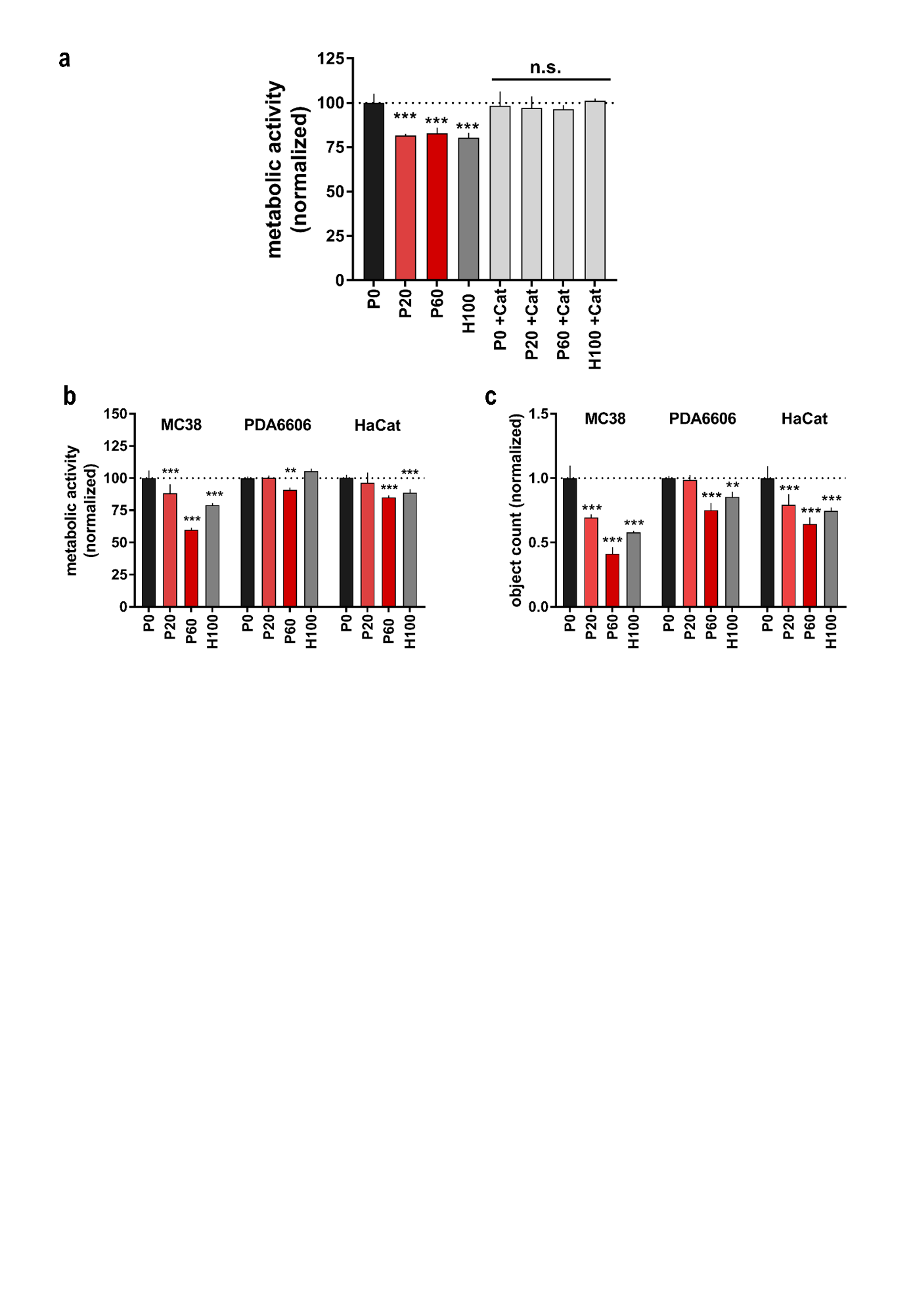


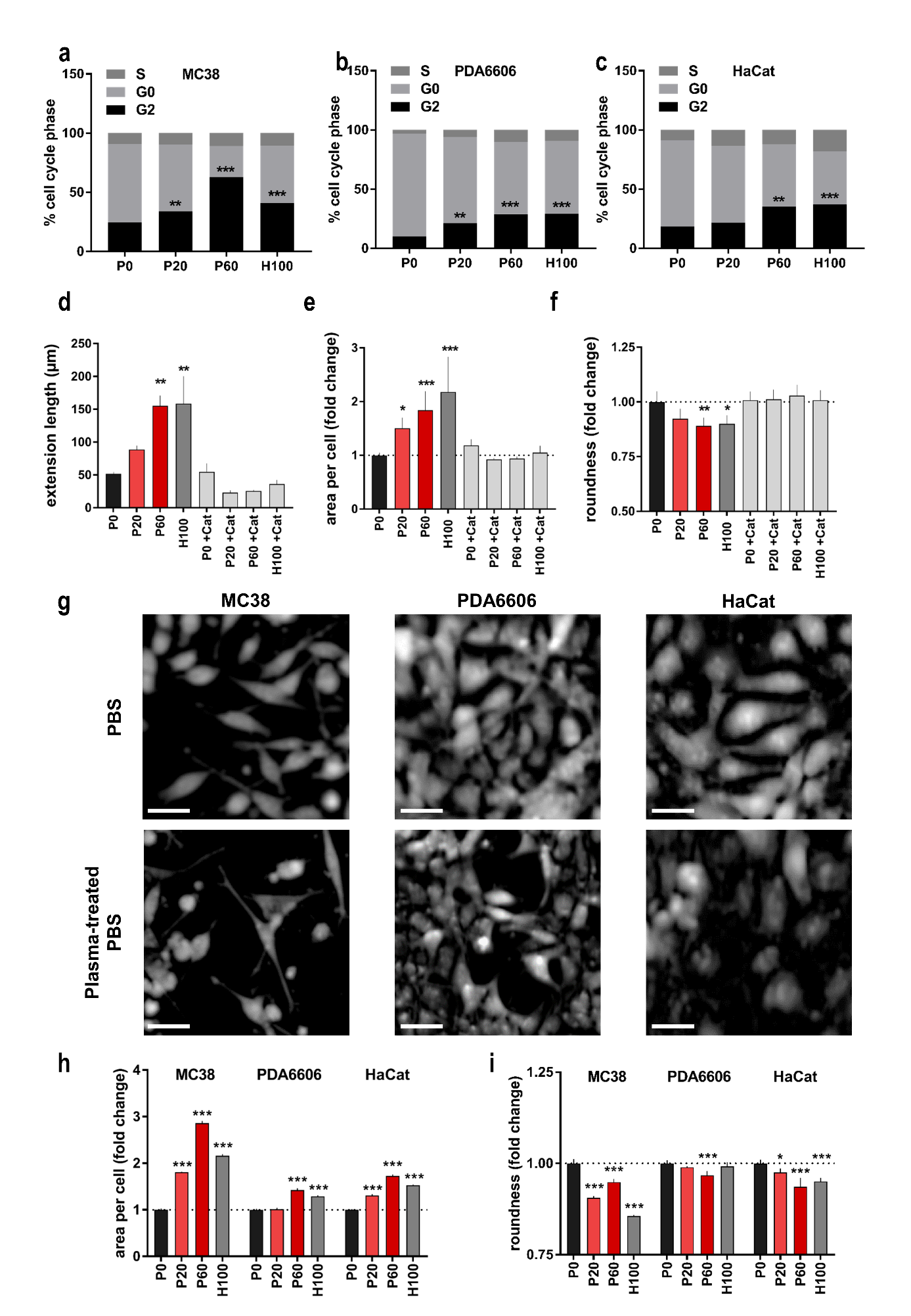


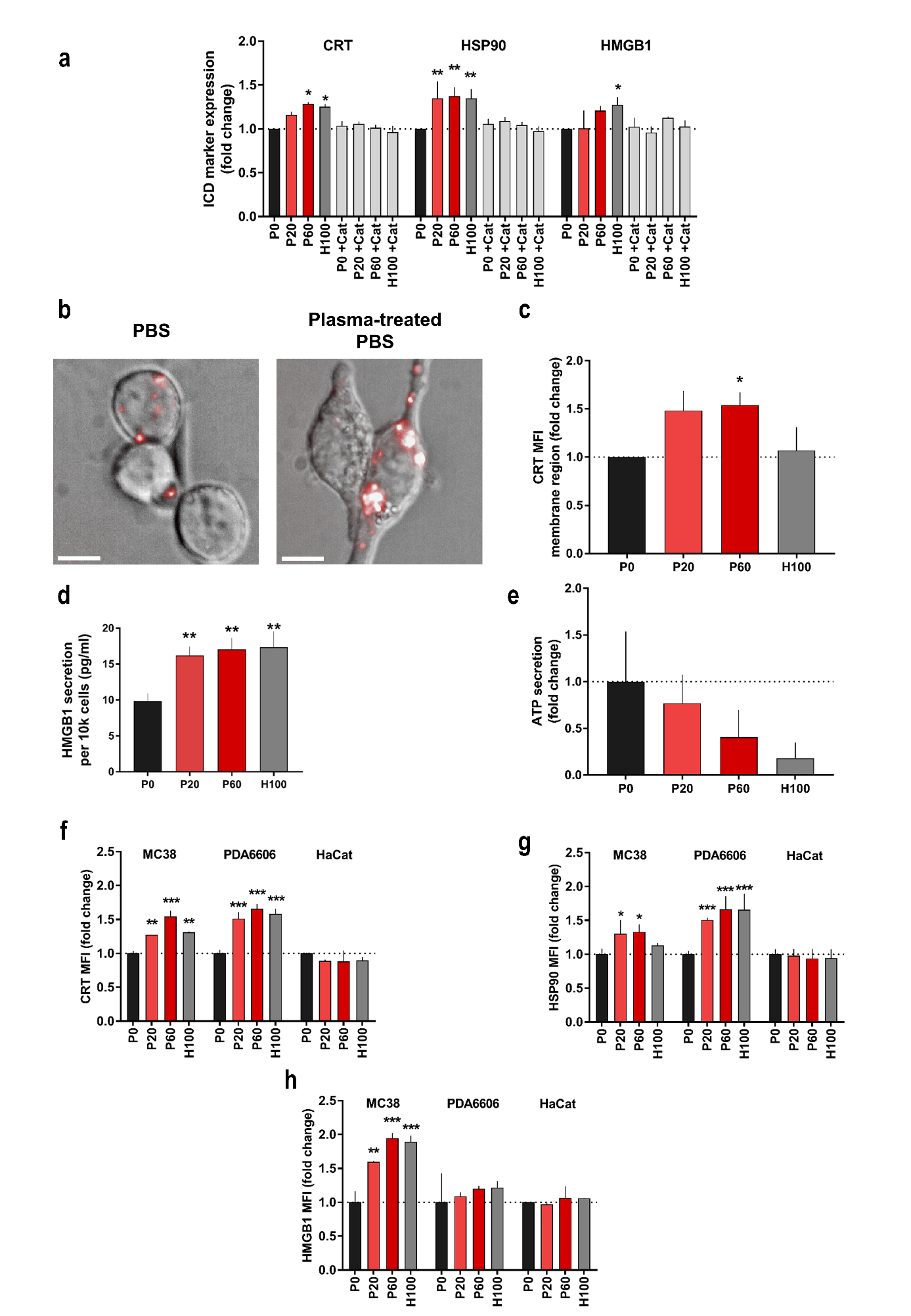


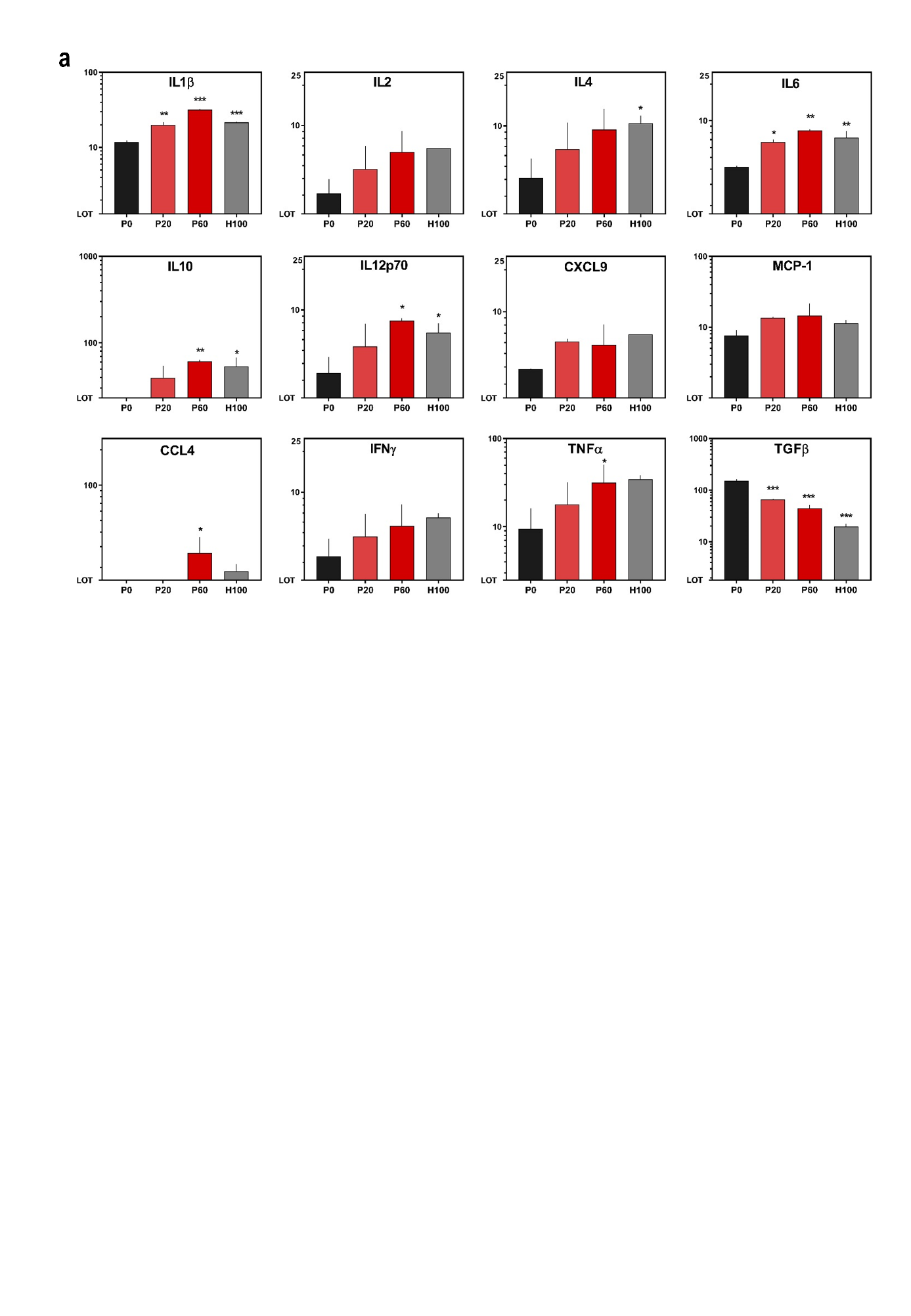

Supplement: Supplementary file 1 — Supplementary Info [file 41598_2018_37169_MOESM1_ESM.docx]
